# State Occupational and Physical Therapy Telehealth Laws and Regulations: A 50-State Survey

**DOI:** 10.5195/ijt.2018.6269

**Published:** 2018-12-11

**Authors:** RANDAL TREY BIERMAN, MEI WA KWONG, CHRISTINE CALOURO

**Affiliations:** CENTER FOR CONNECTED HEALTH POLICY, SACRAMENTO, CA, USA

**Keywords:** Center for Connected Health Policy, Federation of State Boards of Physical Therapy, Occupational therapy, Physical Therapy Licensure Compact, Telehealth, Telemedicine

## Abstract

The Center for Connected Health Policy conducted a scan of current state policy affecting occupational therapy (OT) and physical therapy (PT) practice, supervision, and additional requirements for using telehealth. While most states have established telehealth policies for other health care professions, this 50-state scan shows that many states made some reference to telehealth practice for OT (37 states) and PT (40 states). The states that adopted these policies also tended to adopt them in either law or regulation, but not both, and showed no discernable patterns favoring either. Additionally, eight states included OT and PT within telehealth laws that concurrently apply to multiple health professions. More commonly, states enacted policy within laws or regulations specific to OT and PT. Most policies including limitations on telehealth practice for OT and PT did not appear to create requirements that are more restrictive than what is generally seen in telehealth across all states.

Telehealth is defined as “the use of electronic information and telecommunication technologies to support and promote long-distance clinical health care, patient and professional health-related education, public health and health administration” (Health Resources and Services Administration [HRSA], 2018). Its use is becoming increasingly common by healthcare professionals, including allied healthcare specialties such as occupational and physical therapy. As growth has occurred, many state regulatory agencies have begun to adopt telehealth practice standards. The Center for Connected Health Policy (CCHP) continually tracks telehealth policies across all 50 states and the District of Columbia, and twice a year conducts a scan of the most current telehealth laws, regulations and Medicaid policies. The results from that report are available as a PDF compendium report, as well as an interactive 50 state map on CCHP’s website. Although professional board-related telehealth standards are not a focus of the report, CCHP does take note of adopted professional telehealth policies. In recent years more occupational and physical therapy boards have adopted telehealth regulations (Center for Connected Health Policy, 2018). This has become especially common as private payer laws and Medicaid programs are beginning to allow for reimbursement of allied professionals. To assess the breadth and depth of these standards, CCHP conducted a scan of each state’s occupational therapy (OT) and physical therapy (PT) laws, regulations, and licensing board policies. Specifically, CCHP examined OT and PT practice standards, supervision requirements, and any other limitations on services, modalities, licensing, and establishing the patient-therapist relationship that may apply. These are all common elements that are present in telehealth related laws, regulations, and professional standards in other healthcare professional Boards’ telehealth policies.

Although the terms “telehealth” and “telemedicine” are used interchangeably, the term “telehealth” will be used throughout this paper and is meant to include telemedicine. Additionally, some states have enacted policies allowing for the use of electronic information and telecommunication technologies meeting the definition for telehealth provided by HRSA without using the terms “telehealth,” “telemedicine,” or any variation of these words. Throughout this paper, those policies are referred to as telehealth.

## METHODOLOGY

CCHP surveyed each state for statutes and regulations that included a mention of telehealth or telemedicine in the practice of OT and PT. The results indicate areas where CCHP was unable to find information. Every effort was made to capture the most recent policy language in each state as of August 2018. Although emphasis was placed on regulation and guidance standards, each state’s laws were reviewed to ensure complete and accurate representation of the policy landscape. Searches were made within these sources using the following terms: “telecommunication,” “telehealth,” “tele-,” “telemedicine,” “teletherapy,” “video,” “electronic,” “remote,” “distance,” “distant,” and “supervision.” Although supervision is not directly related to telehealth, it was included in the search due to the quantity of states allowing for supervision via telecommunications, which could include live audio and video modalities. Laws and regulations containing the search terms were examined for relevance to the eight subject areas listed below.

Policies solely referencing education requirements obtainable through video or electronic means were not included as those are not directly related to service delivery. Adoption of the Physical Therapy Licensure Compact (PTLC) was noted under licensing requirements as it allows providers licensed within a compact state to practice in any other compact state through the use of telehealth (The Federation of State Boards of Physical Therapy [FSBPT], 2018). Additionally, OT and PT state board websites were scanned as policies or guidance are sometimes released through other channels.

At least two researchers surveyed each state’s laws and regulations to ensure all relevant information was included. CCHP predetermined eight specific telehealth-related policy areas which frequently appear in professional board documents and occur in technical assistance discussions provided by CCHP regarding both state and federal telehealth-related policy. These areas were then modified based on common trends identified during the initial policy scan. These policy areas, and an additional category for other information not accounted for elsewhere, were used to categorize the relevant information and indicate the topics appearing most frequently. These specific areas are:

- Telehealth/Telemedicine/Telecommunications Definitions
- Location-Type of Site/Geography
- Type of Service
- Supervision
- Informed Consent
- Licensing
- Patient-Provider-Relationship/In-Person Exam Required
- Other

## RESULTS

States overall showed no discernable patterns for using either statutes or regulations for telehealth policy applying to OT and PT. Twenty states had telehealth regulations for OT while 24 had them for PT. Seventeen states had laws for OT while 34 had laws for PT. The higher number of PT laws compared to OT is due to the PTLC which had been adopted into law by 21 states at the time of the scan. Eleven of the states in the PTLC had not passed any other telehealth-related laws, meaning that 23 states had some kind of telehealth-related PT law in addition to or aside from the PTLC.

Thirteen states did not have any telehealth-related OT policy and nine had none for PT. The PTLC was not considered for this number as it is not, in isolation, specifically related to telehealth. Eight states included OT and PT within laws that concurrently apply to multiple health professions. Many policies addressed the use of telecommunications to facilitate the supervision of therapist assistants. Ten states addressed only supervision in their telehealth policies for OT while 13 only addressed supervision for PT. When not including states that only addressed supervision, 27 states had some policy allowing the use of telehealth for patient-to-provider or provider-to-provider consultations for OT and 28 states did so for PT. CCHP noted that eight states with policies for both OT and PT had telehealth laws enacted to cover multiple medical professions in which both OT and PT were included.

CCHP also noted that many states enacted telehealth-related policy without using the words “telehealth,” “telemedicine,” or use of the prefix “tele-” in words such as “telerehabilitation.” Out of the 47 states that included policy for either OT or PT, 25 used one of these terms while 22 only used other terms such as “telecommunications” and “electronic” to connote telehealth services. Policies addressing supervision via telecommunications never used the prior terms, always opting for “telecommunication” or “electronic.” Not including the states that only address supervision, only 9 states did not use the terms “telehealth,” “telemedicine,” or “telerehabilitation” when referring to telecommunication-based patient-to-provider or provider-to-provider consultations.

Thirty-four states that allowed telehealth to be used in OT and PT permitted the use of live video; 23 for OT and 26 for PT. However, these numbers are reduced to 14 and 25, respectively, when considering that eight of these states made reference to live video only for supervising therapist assistants. Twelve states allowed for the use of store-and-forward and seven of these were states that included OT and PT within policies concurrently covering multiple health professions. Nevada was the only state to include OT and PT in a policy covering multiple professions that did not explicitly include store-and-forward (Nev. Rev. Stat. § 629.515). Only four states permitted the use of remote patient monitoring, three of which were states with broad policies. Ohio was the only state without a broad policy to explicitly allow remote monitoring for PT.

Most states did not place additional requirements on providers using telehealth beyond what is otherwise required. As an example, few states indicate patient-provider-relationship requirements. While in many instances it may be dependent upon the nature of the consultation, Arkansas and Idaho explicitly stated that telehealth may be used to establish a patient-provider-relationship for both OT and PT while Virginia stated it is necessary for PT (ACA § 17-80-403; Idaho Code Ann. § 54-5705; VA Board of Physical Therapy, 2018). Additionally, only New Jersey and Rhode Island explicitly required providers to establish a patient-provider-relationship prior to the delivery of services via telehealth (N.J. Rev. Stat. § 45:1-62; RI Department of Health, 2018). No state placed additional geographic restrictions on the use of telehealth for OT or PT.

## DISCUSSION

While most states currently have established policy regarding telehealth practice requirements for primary care providers, those policies sometimes do not include allied professional providers. However, the findings here show that many states’ professional boards are creating their own telehealth policies, particularly OT and PT boards. Similar to what is observed in telehealth policies for other health professions, a uniform approach to establishing policies for OT and PT was not followed across states or professional boards. While many OT and PT professional boards enacted policy for telehealth, nearly as many states have instead adopted laws. Additionally, while there appeared to be some trends regarding terminology and restrictions to specific telehealth modalities, the overall requirements and limitations on OT and PT varied across states. This may be due to a tendency for states to follow pre-existing policy within other specialties in their state or political and economic variances between states which impacts telehealth policies overall. Similar trends have been noted in CCHP’s ongoing telehealth policy tracking. This is further supported by the similarities between OT and PT specific telehealth practice requirements and those of other boards in each state. Although most states are not as comprehensive in their OT or PT policies as they are with other professions, this may be beneficial to telehealth practice in some cases, as fewer requirements may result in greater ease of use among providers.

Many states also used the terms “telecommunications” and “electronic” to refer to technology-enabled communications that include modalities traditionally considered to be telehealth. This is similar to changes made to the 2019 Medicare Physician Fee Schedule, where the terms “telehealth” and “telemedicine” were not used when referring to some technology-enabled services, thereby allowing those services to not be affected by federal statutory limitations on the use of telehealth in Medicare (Center for Medicare & Medicaid Services [CMS], 2018). While a direct correlation cannot be drawn between the two in this research, this may hold implications for the future of telehealth, which may forego use of typical terminology in favor of broader references to technology-enabled care for policies that reflect technologies’ role as just another tool to deliver healthcare.

## SUMMARY

The use of telehealth is becoming more common among allied health professions such as OT and PT. Twenty-seven states have enacted at least one policy allowing patient-to-provider or provider-to-provider services in OT and 28 have done so for PT. The requirements and restrictions on the use of telehealth for both professions varies across all states, however few place additional burdens on providers beyond what is otherwise expected for the delivery of services. This means that OT and PT providers in states which allow them to utilize telehealth to deliver services often may do so to their best clinical judgement and are not restricted to geographic or administrative requirements that exclude some populations from eligibility for telehealth. Just over half of all states provide policy for either OT or PT, though this number may increase as the policy landscape changes at both federal and state levels.

## Appendix

**Table:**

Alabama	
Occupational Therapy	Physical Therapy
Telehealth/Telemedicine/Telecommunications Definition	
No reference found.	No reference found.
Modality	
Telephone and electronic communication are allowed for supervision. ***Source:******AL Admin. Code, Reg. 625-X-8-.01***	No reference found.
Location- Type of site/Geography	
No reference found.	No reference found.
Type of Service	
No reference found.	No reference found.
Supervision	
Alabama allows general supervision via telephone and electronic communication. Direct and close supervision require the supervising occupational therapist to be physically in the same location as the assistant being supervised. ***Source:******AL Admin. Code, Reg. 625-X-8-.01***	No reference found.
Informed Consent	
No reference found.	No reference found.
Patient-Provider-Relationship/In-Person Exam Required	
No reference found.	No reference found.
Licensing	
No reference found.	No reference found.
Other	
No reference found.	No reference found.

**Table:**

Alaska	
Occupational Therapy	Physical Therapy
Telehealth/Telemedicine/Telecommunications Definition	
Telerehabilitation means the practice of therapy by a person licensed as a therapist under AS 08.84 and this chapter using an interactive telecommunication system. Interactive Telecommunications System means: Audio and video equipment that permits a two-way, real time communication between a therapist licensed in Alaska and a patient who is located at a distant site in the state which is not in close proximity to the therapist Does not include: ○ Electronic mail ○ Facsimile machine ○ Telephone ***Source:******12 AAC 54.990(6)***	Telerehabilitation means the practice of therapy by a person licensed as a therapist under AS 08.84 and this chapter using an interactive telecommunication system. Interactive Telecommunications System means: Audio and video equipment that permits a two-way, real time communication between a therapist licensed in Alaska and a patient who is located at a distant site in the state which is not in close proximity to the therapist Does not include: ○ Electronic mail ○ Facsimile machine ○ Telephone ***Source:******12 AAC 54.990(6)***
Modality	
Occupational therapists are permitted to use interactive telecommunications systems. This would include live video and audio. Use of electronic mail, facsimile machine, and telephone are not permitted. ***Source:******12 AAC 54.990***	Physical therapists are permitted to use interactive telecommunications systems. This would include live video and audio. Use of electronic mail, facsimile machine, and telephone are not permitted. ***Source:******12 AAC 54.990***
Location- Type of site/Geography	
Occupational therapists must be physically located in the state of Alaska while conducting telerehabilitation. ***Source:******12 AAC 54.825***	Physical therapists must be physically located in the state of Alaska while conducting telerehabilitation. ***Source:******12 AAC 54.530***
Type of Service	
No reference found.	No reference found.
Supervision	
No reference found.	No reference found.
Informed Consent	
No reference found.	No reference found.
Patient-Provider-Relationship/In-Person Exam Required	
Occupational therapists may use interactive telecommunications to conduct one-on-one consultations, including initial evaluation. ***Source:******12 AAC 54.530(4)***	Physical therapists may use interactive telecommunications to conduct one-on-one consultations, including initial evaluation. ***Source:******12 AAC 54.530(4)***
Licensing	
Occupational therapists must be licensed in Alaska to provide telehealth services to patients in the state. ***Source:******12 AAC 54.530(b)***	Physical therapists must be licensed in Alaska to provide telehealth services to patients in the state. ***Source:******12 AAC 54.530(b)***
Other	
Occupational therapists must maintain the same ethical conduct and integrity as in-person rehabilitation. They may conduct one-on-one consultations, including initial evaluations, and must provide and ensure appropriate client confidentiality and HIPAA compliance. ***Source:******12 AAC 54.825(2)***	Physical therapists must maintain the same ethical conduct and integrity as in-person rehabilitation. They may conduct one-on-one consultations, including initial evaluations, and must provide and ensure appropriate client confidentiality and HIPAA compliance. ***Source:******12 AAC 54.530(2)***

**Table:**

Arizona	
Occupational Therapy	Physical Therapy
Telehealth/Telemedicine/Telecommunications Definition	
No reference found.	No reference found.
Modality	
Telephone is permitted to be used for supervision within certain conditions. ***Source:******AAC R4-43-101***	Telecommunications may be used for some supervision. This would include live audio and video and telephone. ***Source:******ARS § 32-2001***
Location- Type of site/Geography	
No reference found.	No reference found.
Type of Service	
No reference found.	No reference found.
Supervision	
Arizona allows a supervising occupational therapist to provide general supervision to an occupational therapist assistant via telephone or written communication as long as the supervising OT has face-to-face contact with the OTA at least once every 30-calendar days per client basis while on the premises. ***Source:******AAC R4-43-101***	Arizona allows a supervising physical therapist to provide general supervision to a physical therapist assistant via telecommunications. Telecommunications may not be used for other forms of supervision. ***Source:******ARS § 32-2001***
Informed Consent	
No reference found.	No reference found.
Patient-Provider-Relationship/In-Person Exam Required	
No reference found.	No reference found.
Licensing	
No reference found.	Arizona is part of the Physical Therapy Compact. Physical therapists licensed through the compact are permitted to practice in other states participating in the compact or in Arizona if they are located in another state. ***Source:******ARS § 32-2053***
Other	
No reference found.	No reference found.

**Table:**

Arkansas	
Occupational Therapy	Physical Therapy
Telehealth/Telemedicine/Telecommunications Definition	
“Telemedicine” means the use of electronic information and communication technology to deliver healthcare services, including without limitation the assessment, diagnosis, consultation, treatment, education, care management, and self-management of a patient. “Telemedicine” includes store-and-forward technology and remote patient monitoring. ***Source:******ACA § 17-80-402***	“Telecommunication” means the electronic transmission, conveyance, or routing of voice, data, audio, video, or any other information or signals to a point or between or among points. ***Source:******ACA § 17-93-102*** “Telemedicine” means the use of electronic information and communication technology to deliver healthcare services, including without limitation the assessment, diagnosis, consultation, treatment, education, care management, and self-management of a patient. “Telemedicine” includes store-and-forward technology and remote patient monitoring. ***Source:******ACA § 17-80-402***
Modality	
Telemedicine is defined as including store-and-forward and remote patient monitoring. Occupational therapists may also use live audio and video. ***Source:******ACA § 17-80-402 & ACA § 17-80-404*** “Remote patient monitoring” means the use of synchronous or 1electronic information and communication technology to collect personal health information and medical data from a patient at an originating site that is transmitted to a healthcare professional at a distant site for use in the treatment and management of medical conditions that require frequent monitoring. “Store-and-forward technology” means the asynchronous transmission of a patient’s medical information from a healthcare professional at an originating site to a healthcare professional at a distant site. ***Source:******ACA § 17-80-402***	Telemedicine is defined as including store-and-forward and remote patient monitoring. Physical therapists may also use live audio and video. ***Source:******ACA § 17-80-402 & ACA § 17-80-404*** “Remote patient monitoring” means the use of synchronous or asynchronous electronic information and communication technology to collect personal health information and medical data from a patient at an originating site that is transmitted to a healthcare professional at a distant site for use in the treatment and management of medical conditions that require frequent monitoring. “Store-and-forward technology” means the asynchronous transmission of a patient’s medical information from a healthcare professional at an originating site to a healthcare professional at a distant site. ***Source:******ACA § 17-80-402***
Location- Type of site/Geography	
“Distant site” means the location of the healthcare professional delivering services through telemedicine at the time the services are provided. “Originating site” means a site at which a patient is located at the time healthcare services are provided to him or her by means of telemedicine. ***Source:******ACA § 17-80-402***	“Distant site” means the location of the healthcare professional delivering services through telemedicine at the time the services are provided. “Originating site” means a site at which a patient is located at the time healthcare services are provided to him or her by means of telemedicine. ***Source:******ACA § 17-80-402***
Type of Service	
No reference found.	“Consultation by means of telecommunication” means the rendering of a professional opinion, expert opinion, or advice by a physical therapist to another physical therapist or health care provider through telecommunication technology. “Consultation by means of telecommunication” includes the review or transfer of patient records or related information through telecommunication technology ***Source:******ACA § 17-93-102***
Supervision	
No reference found.	A physical therapist providing supervision is not required to be on-site, but must be “readily available for consultation” which means “the ability to be reached in-person or by telecommunications within 30 minutes.” ***Source:******ACR R71-00-001***
Informed Consent	
Occupational therapists must follow applicable state and federal law, rules, and regulations for informed consent. ***Source:******ACA § 17-80-404***	Physical therapists must follow applicable state and federal law, rules, and regulations for informed consent. ***Source:******ACA § 17-80-404***
Patient-Provider-Relationship/In-Person Exam Required	
A healthcare professional may not provide telemedicine services to a patient located in Arkansas unless a professional relationship exists between the healthcare professional and the patient or the healthcare professional otherwise meets the requirements of a professional relationship. The existence of a professional relationship is not required in the following circumstances: Emergency situations where the life or health of the patient is in danger or imminent danger; or Simply providing in formation of a generic nature, not meant to be specific to an individual patient. If the establishment of a professional relationship is permitted via telemedicine in the professional relationship requirements (below), telemedicine may be used to establish the professional relationship only for situations in which the standard of care does not require an in-person encounter. The following may not be used to establish a professional relationship: An internet questionnaire; An email message; Patient-generated medical history; Audio-only communication, including without limitation interactive audio; Text messaging; A facsimile machine; or Any combination thereof. ***Source:******ACA § 17-80-403*** A professional relationship is established: After a healthcare professional therapist has conducted an in-person examination and is available to provide appropriate follow-up care, when necessary, at medically necessary intervals; The healthcare professional personally knows the patient and the patient’s relevant health status through an ongoing personal or professional relationship and is available to provide appropriate follow-up care, when necessary, at medically necessary intervals; The treatment is provided by a healthcare professional in consultation with, or upon referral by, another healthcare professional who has an ongoing relationship with the patient and who has agreed to supervise the patient’s treatment, including follow-up care; An on-call or cross-coverage arrangement exists with the patient’s regular treating healthcare professional or another healthcare professional who has established a professional relationship with the patient; A relationship exists in other circumstances as defined by rule of the Arkansas State Medical Board for healthcare professionals under its jurisdiction and their patients; or A relationship exists in other circumstances as defined by rule of a licensing or certification board for other healthcare professionals under the jurisdiction of the appropriate board and their patients if the rules are no less restrictive than the rules of the Arkansas State Medical Board ***Source:******ACA § 17-80-402***	A healthcare professional may not provide telemedicine services to a patient located in Arkansas unless a professional relationship exists between the healthcare professional and the patient or the healthcare professional otherwise meets the requirements of a professional relationship. The existence of a professional relationship is not required in the following circumstances: Emergency situations where the life or health of the patient is in danger or imminent danger; or Simply providing in formation of a generic nature, not meant to be specific to an individual patient. If the establishment of a professional relationship is permitted via telemedicine in the professional relationship requirements (below), telemedicine may be used to establish the professional relationship only for situations in which the standard of care does not require an in-person encounter. The following may not be used to establish a professional relationship: An internet questionnaire; An email message; Patient-generated medical history; Audio-only communication, including without limitation interactive audio; Text messaging; A facsimile machine; or Any combination thereof. ***Source:******ACA § 17-80-403*** A professional relationship is established: After a healthcare professional therapist has conducted an in-person examination and is available to provide appropriate follow-up care, when necessary, at medically necessary intervals; The healthcare professional personally knows the patient and the patient’s relevant health status through an ongoing personal or professional relationship and is available to provide appropriate follow-up care, when necessary, at medically necessary intervals; The treatment is provided by a healthcare professional in consultation with, or upon referral by, another healthcare professional who has an ongoing relationship with the patient and who has agreed to supervise the patient’s treatment, including follow-up care; An on-call or cross-coverage arrangement exists with the patient’s regular treating healthcare professional or another healthcare professional who has established a professional relationship with the patient; A relationship exists in other circumstances as defined by rule of the Arkansas State Medical Board for healthcare professionals under its jurisdiction and their patients; or A relationship exists in other circumstances as defined by rule of a licensing or certification board for other healthcare professionals under the jurisdiction of the appropriate board and their patients if the rules are no less restrictive than the rules of the Arkansas State Medical Board ***Source:******ACA § 17-80-402***
Licensing	
A licensing or certification board shall not permit the use of telemedicine in a manner that is less restrictive than the use of telemedicine authorized by the Arkansas State Medical Board. ***Source:******ACA § 17-80-404*** Occupational therapists must be licensed or certified in Arkansas to provide telehealth services to patients in the state. ***Source:******ACA § 17-80-404***	A person is exempt from the licensing requirements if she is “A physical therapist who is licensed in another jurisdiction of the United States if the physical therapist is providing consultation by means of telecommunication to a physical therapist licensed by the board under this chapter;” ***Source:******ACA § 17-93-301*** A licensing or certification board shall not permit the use of telemedicine in a manner that is less restrictive than the use of telemedicine authorized by the Arkansas State Medical Board. ***Source:******ACA § 17-80-404*** Physical therapists must be licensed or certified in Arkansas to provide telehealth services to patients in the state. ***Source:**** ACA § 17-80-404*
Other	
Services provided via telemedicine shall be held to the same standard of care as healthcare services provided in person. ***Source:******ACA § 17-80-404***	No reference found.

**Table:**

California	
Occupational Therapy	Physical Therapy
Telehealth/Telemedicine/Telecommunications Definition	
“Telehealth” means the mode of delivering health care services and public health via information and communication technologies to facilitate the diagnosis, consultation, treatment, education, care management, and self-management of a patient’s health care while the patient is at the originating site and the health care provider is at a distant site. Telehealth facilitates patient self-management and caregiver support for patients and includes synchronous interactions and asynchronous store and forward transfers. ***Source:******Cal. BPC §2290.5***	“Telehealth” means the mode of delivering health care services and public health via information and communication technologies to facilitate the diagnosis, consultation, treatment, education, care management, and self-management of a patient’s health care while the patient is at the originating site and the health care provider is at a distant site. Telehealth facilitates patient self-management and caregiver support for patients and includes synchronous interactions and asynchronous store and forward transfers. ***Source:******Cal. BPC §2290.5***
Modality	
Occupational therapists are permitted to use synchronous and store-and-forward telehealth. ***Source:******Cal. BPC §2290.5*** “Synchronous interaction” means a real-time interaction between a patient and a health care provider located at a distant site. “Asynchronous store and forward” means the transmission of a patient’s medical information from an originating site to the health care provider at a distant site without the presence of the patient. ***Source:******Cal. BPC §2290.5***	Physical therapists are permitted to use synchronous and store-and-forward telehealth. ***Source:******Cal. BPC §2290.5*** “Synchronous interaction” means a real-time interaction between a patient and a health care provider located at a distant site. “Asynchronous store and forward” means the transmission of a patient’s medical information from an originating site to the health care provider at a distant site without the presence of the patient. ***Source:******Cal. BPC §2290.5***
Location- Type of site/Geography	
“Distant site” means a site where a health care provider who provides health care services is located while providing these services via a telecommunications system. ***Source:******Cal. BPC §2290.5***	“Distant site” means a site where a health care provider who provides health care services is located while providing these services via a telecommunications system. ***Source:******Cal. BPC §2290.5***
Type of Service	
Occupational therapists may provide services consistent with California regulations and that comply with the California Occupational Therapy Practice Act. ***Source:******Cal. Code Regs., tit. 16, §4172***	No reference found.
Supervision	
No reference found.	No reference found.
Informed Consent	
An occupational therapist shall inform the patient or client about occupational therapy services via telehealth and obtain verbal or written consent prior to delivering those services. ***Source:******Cal. Code Regs., tit. 16, §4172***	A physical therapist must inform the patient about the use of telehealth and obtain verbal or written consent from the patient prior to delivering services via telehealth. ***Source:******Cal. BPC §2290.5***
Patient-Provider-Relationship/In-Person Exam Required	
No reference found.	No reference found.
Licensing	
An occupational therapist must be licensed in California to provide telehealth services to patients in the state. ***Source:******Cal. BPC §2290.5***	A physical therapist must be licensed in California to provide telehealth services to patients in the state. ***Source:******Cal. BPC §2290.5***
Other	
An occupational therapist or occupational therapy assistant providing therapy via telehealth must: Adhere to the same standard of care as when providing services via any other mode of delivery; Provide services consistent with California regulations; and Comply with all provisions of the California Occupational Therapy Practice Act, including ethical standards of practice. An occupational therapist shall determine whether an in-person evaluation or in-person interventions are necessary considering: the complexity of the patient’s/client’s condition; his or her own knowledge, skills, and abilities; the nature and complexity of the intervention; the requirements of the practice setting; and the patient’s/client’s context and environment. ***Source:******Cal. Code Regs., tit. 16, §4172***	No reference found.

**Table:**

Colorado	
Occupational Therapy	Physical Therapy
Telehealth/Telemedicine/Telecommunications Definition	
No reference found.	No reference found.
Modality	
No reference found.	No reference found.
Location- Type of site/Geography	
No reference found.	No reference found.
Type of Service	
Occupational therapy can include the use of telehealth pursuant to rules adopted by the director of the Board of Occupational Therapy. ***Source:******Colo. Rev. Stat. § 12-40.5-103***	No reference found.
Supervision	
No reference found.	A physical therapist is not required to be on site for general supervision, but must be available at least via telecommunications. ***Source:******4 Colo. Code Regs. § 732-1-101***
Informed Consent	
No reference found.	No reference found.
Patient-Provider-Relationship/In-Person Exam Required	
No reference found.	No reference found.
Licensing	
No reference found.	Colorado is part of the Physical Therapy Compact. Physical therapists licensed through the compact are permitted to practice in other states participating in the compact or in Colorado if they are located in another state. ***Source:******Colo. Rev. Stat. § 24-60-3702***
Other	
No reference found.	No reference found.

**Table:**

Connecticut	
Occupational Therapy	Physical Therapy
Telehealth/Telemedicine/Telecommunications Definition	
No reference found.	No reference found.
Modality	
No reference found.	No reference found.
Location- Type of site/Geography	
No reference found.	No reference found.
Type of Service	
No reference found.	No reference found.
Supervision	
No reference found.	No reference found.
Informed Consent	
No reference found.	No reference found.
Patient-Provider-Relationship/In-Person Exam Required	
No reference found.	No reference found.
Licensing	
No reference found.	No reference found.
Other	
No reference found.	No reference found.

**Table:**

Delaware	
Occupational Therapy	Physical Therapy
Telehealth/Telemedicine/Telecommunications Definition	
“Telehealth” is the use of electronic communications to provide and deliver a host of health-related information and health-care services including occupational therapy services. “Telemedicine” means a form of telehealth which is the delivery of clinical health-care services by means of real time 2-way audio, visual, or other telecommunications or electronic communications, including the application of secure video conferencing or store and forward transfer technology to provide or support health-care delivery, which facilitate the assessment, diagnosis, consultation, treatment, education, care management and self-management of a patient’s health care by a licensee practicing within his or her scope of practice as would be practiced in-person with a patient and with other restrictions as defined in regulation. ***Source:******Del. C. §24-2002***	“Telehealth,” is the use of electronic communications to provide and deliver a host of health-related information and health-care services, including physical therapy and athletic training-related information and services, over large and small distances. Telehealth encompasses a variety of health care and health promotion activities, including education, advice, reminders, interventions, and monitoring of intervention. ***Source:******18-1400 Del. Admin. Code § 1409******&******Del. C. §24-2602***
Modality	
Occupational therapists may engage in live video and store-and-forward. ***Source:******Del. C. §24-2002***	Physical therapists may use electronic communications to deliver some services and telecommunications to provide supervision. ***Source:******18-1400 Del. Admin. Code § 1409******&******Del. C. §24-2600***
Location- Type of site/Geography	
“Originating site” means a site in Delaware at which a patient is located at the time health-care services are provided to him or her by means of telemedicine or telehealth, unless the term is otherwise defined with respect to the provision in which it is used; provided, however, notwithstanding any other provision of law, insurers and providers may agree to alternative siting arrangements deemed appropriate by the parties. “Distant site” means a site at which a health-care provider legally allowed to practice in the State is located while providing health-care services by means of telemedicine or telehealth. ***Source:******Del. C. §24-2002***	During the telehealth treatment session, the patient shall be located within the borders of the State of Delaware. ***Source:******Del. C. §24-2602***
Type of Service	
Services may be provided through the use of telemedicine in a manner deemed appropriate by regulation. Services also may include participation in telehealth as further defined in regulation. ***Source:******Del. C. §24-2002***	Telehealth encompasses a variety of health care and health promotion activities, including education, advice, reminders, interventions, and monitoring of intervention. ***Source:******18-1400 Del. Admin. Code § 1409***
Supervision	
No reference found.	A supervising physical therapist is required to be accessible via telecommunications to a physical therapist assistant during all work hours of the physical therapist assistant. ***Source:******Del. C. §24-2600*** Subject to supervision requirements, every other supervisory visit may be performed via telehealth with the other alternating visit performed face-to-face. ***Source:******18-1400 Del. Admin. Code § 1409***
Informed Consent	
Before services are provided through telehealth, the licensee shall obtain written, informed consent from the patient, or other appropriate person with authority to make health care treatment decisions for the patient. ***Source:******Del. C. §24-2002***	Before providing telehealth services, a licensee must obtain written, informed consent from a patient, or other appropriate person with authority to make health care treatment decisions for the patient. The informed consent must inform the patient and document acknowledgement of the risk and limitations of: The use of electronic communications in the provision of care; The potential breach of confidentiality, or inadvertent access, of protected health information using electronic communication in the provision of care; and The potential disruption of electronic communication in the use of telehealth. ***Source:******Del. C. §24-2600***
Patient-Provider-Relationship/In-Person Exam Required	
No reference found.	All evaluations, including initial evaluations, and re-evaluations and scheduled discharges shall be performed face-to-face and not through telehealth. ***Source:******Del. C. §24-2600***
Licensing	
Occupational therapists are required to be licensed in Delaware if providing occupational therapy services to a patient located in the state. ***Source:******Del. C. §24-2002***	Physical therapists are required to be licensed in Delaware if providing services to a patient located in the state. ***Source:******Del. C. §24-2605***
Other	
No reference found.	No reference found.

**Table:**

Florida	
Occupational Therapy	Physical Therapy
Telehealth/Telemedicine/Telecommunications Definition	
No reference found.	No reference found.
Modality	
No reference found.	Physical therapists may use telecommunications to provide some supervision. ***Source:******Fla. Admin. Code r. 64B17-6.001***
Location- Type of site/Geography	
No reference found.	A physical therapist providing supervision via telecommunications, must be within the same geographic location as the physical therapy assistant. ***Source:******Fla. Admin. Code r. 64B17-6.001***
Type of Service	
No reference found.	No reference found.
Supervision	
No reference found.	While a physical therapy assistant is delivering services to patients who are not inpatients in a hospital, or who are not in the acute phase of injury or illness, the physical therapist must be accessible at all times via telecommunications. ***Source:******Fla. Admin. Code r. 64B17-6.001***
Informed Consent	
No reference found.	No reference found.
Patient-Provider-Relationship/In-Person Exam Required	
No reference found.	No reference found.
Licensing	
No reference found.	No reference found.
Other	
No reference found.	No reference found.

**Table:**

Georgia	
Occupational Therapy	Physical Therapy
Telehealth/Telemedicine/Telecommunications Definition	
No reference found.	Telehealth is the use of electronic communications to provide and deliver a host of health-related information and health care services including, but not limited to physical therapy related information and services, over large and small distances. Telehealth encompasses a variety of health care and health promotion activities including, but not limited to, education, advice, reminders, interventions, and monitoring of interventions. ***Source:******Georgia State Board of Physical Therapy***
Modality	
No reference found.	No reference found.
Location- Type of site/Geography	
No reference found.	No reference found.
Type of Service	
No reference found.	Telehealth may be used to deliver physical therapy related information and services including, but not limited to, education, advice, reminders, interventions, and monitoring of interventions. ***Source:******Georgia State Board of Physical Therapy***
Supervision	
No reference found.	No reference found.
Informed Consent	
No reference found.	No reference found.
Patient-Provider-Relationship/In-Person Exam Required	
No reference found.	No reference found.
Licensing	
No reference found.	A physical therapist licensed in another jurisdiction of the United States may provide a consultation via telehealth to a physical therapist in the state of Georgia. ***Source:******Georgia State Board of Physical Therapy***
Other	
No reference found.	No reference found.

**Table:**

Hawaii	
Occupational Therapy	Physical Therapy
Telehealth/Telemedicine/Telecommunications Definition	
No reference found.	No reference found.
Modality	
No reference found.	Physical therapists may use telecommunications, such as live video and audio or telephone to provide some supervision. ***Source:******Haw. Code. R. § 16-110-4***
Location- Type of site/Geography	
No reference found.	No reference found.
Type of Service	
No reference found.	No reference found.
Supervision	
No reference found.	Hawaii requires supervising physical therapists to be available at all times via telecommunications when not on site and be able to be on site within two hours if needed in-person. ***Source:******Haw. Code. R. § 16-110-4***
Informed Consent	
No reference found.	No reference found.
Patient-Provider-Relationship/In-Person Exam Required	
No reference found.	No reference found.
Licensing	
No reference found.	No reference found.
Other	
No reference found.	No reference found.

**Table:**

Idaho	
Occupational Therapy	Physical Therapy
Telehealth/Telemedicine/Telecommunications Definition	
“Telehealth services” means health care services provided by a provider to a person through the use of electronic communications, information technology, asynchronous store and forward transfer or synchronous interaction between a provider at a distant site and a patient at an originating site. Such services include, but are not limited to, clinical care, health education, home health and facilitation of self-managed care and caregiver support. ***Source:******Idaho Code Ann. § 54-5703***	“Telehealth services” means health care services provided by a provider to a person through the use of electronic communications, information technology, asynchronous store and forward transfer or synchronous interaction between a provider at a distant site and a patient at an originating site. Such services include, but are not limited to, clinical care, health education, home health and facilitation of self-managed care and caregiver support. ***Source:******Idaho Code Ann. § 54-5703***
Modality	
Occupational therapists are permitted to use live video and audio or audio transmission as well as store-and-forward. ***Source:******Idaho Code Ann. § 54-5703*** Treatment based solely on an online questionnaire does not constitute an acceptable standard of care. ***Source:******Idaho Code Ann. § 54-5706***	Physical therapists are permitted to use live video and audio or audio transmission as well as store-and-forward. ***Source:******Idaho Code Ann. § 54-5703*** Treatment based solely on an online questionnaire does not constitute an acceptable standard of care. ***Source:******Idaho Code Ann. § 54-5706*** Physical therapists may use telecommunications including live video and audio or telephone when providing supervision. ***Source:******Idaho Admin. Code r. 24.13.01***
Location- Type of site/Geography	
“Distant site” means the site at which a provider delivering telehealth services is located at the time the service is provided. “Originating site” means the location of a patient at the time telehealth services are provided. ***Source:******Idaho Code Ann. § 54-5703***	“Distant site” means the site at which a provider delivering telehealth services is located at the time the service is provided. “Originating site” means the location of a patient at the time telehealth services are provided. ***Source:******Idaho Code Ann. § 54-5703***
Type of Service	
Services include, but are not limited to, clinical care, health education, home health and facilitation of self-managed care and caregiver support. ***Source:******Idaho Code Ann. § 54-5703***	Services include, but are not limited to, clinical care, health education, home health and facilitation of self-managed care and caregiver support. ***Source:******Idaho Code Ann. § 54-5703***
Supervision	
No reference found.	Idaho requires supervising physical therapists to be available by means of telecommunications if not available in-person when assisting a physical therapist assistant. ***Source:******Idaho Admin. Code r. 24.13.01***
Informed Consent	
A patient’s informed consent for the use of telehealth services shall be obtained as required by applicable law. ***Source:******Idaho Code Ann. § 54-5708***	A patient’s informed consent for the use of telehealth services shall be obtained as required by applicable law. ***Source:******Idaho Code Ann. § 54-5708***
Patient-Provider-Relationship/In-Person Exam Required	
A provider offering telehealth services in his or her practice may establish a provider-patient relationship by use of two-way audio and visual interaction if the applicable Idaho community standard of care is satisfied. ***Source:******Idaho Code Ann. § 54-5705***	A provider offering telehealth services in his or her practice may establish a provider-patient relationship by use of two-way audio and visual interaction if the applicable Idaho community standard of care is satisfied. ***Source:******Idaho Code Ann. § 54-5705***
Licensing	
Occupational therapists are required to be licensed in Idaho if providing telehealth services to a patient located in the state. ***Source:******Idaho Code Ann. § 54-5703***	Physical therapists are required to be licensed in Idaho if providing telehealth services to a patient located in the state. ***Source:******Idaho Code Ann. § 54-5703***
Other	
Prior to providing treatment, an occupational therapist is required to document a patient’s relevant clinical history and current symptoms to establish the diagnosis and identify underlying conditions and contraindications to the treatment recommended. ***Source:******Idaho Code Ann. § 54-5706*** Occupational therapists are required to record the use of telehealth services in a patient’s medical records. ***Source:******Idaho Code Ann. § 54-5711***	Prior to providing treatment, a physical therapist is required to document a patient’s relevant clinical history and current symptoms to establish the diagnosis and identify underlying conditions and contraindications to the treatment recommended. ***Source:******Idaho Code Ann. § 54-5706*** Physical therapists are required to record the use of telehealth services in a patient’s medical records. ***Source:******Idaho Code Ann. § 54-5711***

**Table:**

Illinois	
Occupational Therapy	Physical Therapy
Telehealth/Telemedicine/Telecommunications Definition	
Telehealth is defined as services provided via technology or telecommunication methods. ***Source:******225 Ill. Comp. Stat. § 75/2***	No reference found.
Modality	
Occupational therapy, including supervision under certain conditions, may be provided via telecommunication, which would include live video and audio and telephone. ***Source:******225 Ill. Comp. Stat. § 75/2***	No reference found.
Location- Type of site/Geography	
No reference found.	No reference found.
Type of Service	
Occupational therapy may be provided via telehealth if the same standard of care is met as would be present during an in-person visit. ***Source:******225 Ill. Comp. Stat. § 75/2***	No reference found.
Supervision	
Illinois supervising occupational therapists may provide supervision via electronic communication, telecommunication, or technology to an occupational therapy assistant with less than one year of experience in a practice or who is learning new skills as long as they provide 5% of supervision as face-to-face consultation. Electronic communication, telecommunication, or technology may contribute towards up to 3% of the required 5% direct supervision for supervision of occupational therapy assistants with more than one year of experience in their current practice. ***Source:******Ill. Admin. Code tit. 68, pt. 1315***	No reference found.
Informed Consent	
No reference found.	No reference found.
Patient-Provider-Relationship/In-Person Exam Required	
No reference found.	No reference found.
Licensing	
Occupational therapists must be licensed in Illinois to provide telehealth services to a patient located in the state unless they are licensed in another state, with at least as restrictive licensure requirements as Illinois, for no more than 60 days in a calendar year. ***Source:******225 Ill. Comp. Stat. § 75/3***	No reference found.
Other	
No reference found.	No reference found.

**Table:**

Indiana	
Occupational Therapy	Physical Therapy
Telehealth/Telemedicine/Telecommunications Definition	
No reference found.	No reference found.
Modality	
No reference found.	Telecommunications devices for the deaf (TDD) may be used in consultations between physical therapists and physical therapy assistants under certain circumstances. ***Source:******844 Ind. Admin. Code 6-1-2***
Location- Type of site/Geography	
No reference found.	No reference found.
Type of Service	
No reference found.	No reference found.
Supervision	
No reference found.	A consultation between a supervising physical therapist or physician and a physical therapist’s assistant may be conducted via telecommunications device for the deaf (TDD), if the consultation is concerning patient care. ***Source:******844 Ind. Admin. Code 6-1-2***
Informed Consent	
No reference found.	No reference found.
Patient-Provider-Relationship/In-Person Exam Required	
No reference found.	No reference found.
Licensing	
No reference found.	No reference found.
Other	
No reference found.	No reference found.

**Table:**

Iowa	
Occupational Therapy	Physical Therapy
Telehealth/Telemedicine/Telecommunications Definition	
A telehealth visit means the provision of occupational therapy services by a licensee to a patient using technology where the licensee and the patient are not at the same physical location for the occupational therapy session. ***Source:******Iowa Admin. Code r. 645-208.3(1)***	A telehealth visit means the provision of physical therapy services by a licensee to a patient using technology where the licensee and the patient are not at the same physical location for the physical therapy session. ***Source:******Iowa Admin. Code r. 645-201.3(1)***
Modality	
Occupational therapists may use live audio and video. Store-and-forward and non-real-time communication may be used to prepare for an occupational therapy session or to communicate with a patient between occupational therapy sessions. ***Source:******Iowa Admin. Code r. 645-208.3(2)***	Physical therapists may use live audio and video. Store-and-forward and other non-real-time communication may be used to prepare for the physical therapy session or to communicate with a patient in between sessions. ***Source:******Iowa Admin. Code r. 645-201.3(2)***
Location- Type of site/Geography	
No reference found.	No reference found.
Type of Service	
The occupational therapist using telehealth must meet the same standard of care as when providing in-person occupational therapy and may only provide services in the areas of competence in which they are proficient. ***Source:******Iowa Admin. Code r. 645-208.3(6)***	The physical therapist using telehealth must meet the same standard of care as when providing in-person physical therapy and may only provide services in the areas of competence in which they are proficient. ***Source:******Iowa Admin. Code r. 645-201.3(6)***
Supervision	
No reference found.	No reference found.
Informed Consent	
Prior to the first telehealth visit, the occupational therapist must obtain informed consent from the patient specific to the occupational therapy services that will be provided in the telehealth visit. The informed consent must inform the patient of the following, at minimum: The risks and limitations of the use of technology to provide occupational therapy services; The potential for unauthorized access to protected health information; and The potential for disruption of technology during a telehealth visit. ***Source:******Iowa Admin. Code r. 645-208.3(5)***	Prior to the first telehealth visit, the physical therapist must obtain informed consent from the patient specific to the physical therapy services that will be provided in the telehealth visit. The informed consent must inform the patient of the following, at minimum: The risks and limitations of the use of technology to provide physical therapy services; The potential for unauthorized access to protected health information; and The potential for disruption of technology during a telehealth visit. ***Source:******Iowa Admin. Code r. 645-201.3(5)***
Patient-Provider-Relationship/In-Person Exam Required	
No reference found.	No reference found.
Licensing	
Occupational therapists must be licensed in the state of Iowa if providing telehealth services to patients located within the state. ***Source:******Iowa Admin. Code r. 645-208.3(4)***	Iowa is part of the Physical Therapy Licensure Compact. Physical therapists licensed through the compact may provide services in other compact states and in Iowa. ***Source:******Iowa Code § 147C.1***
Other	
No reference found.	No reference found.

**Table:**

Kansas	
Occupational Therapy	Physical Therapy
Telehealth/Telemedicine/Telecommunications Definition	
No reference found.	No reference found.
Modality	
Telephone, electronic mail, text messaging, and written communication may be used for supervision under certain conditions. ***Source:******Kan. Admin. Regs. § 100-54-12***	No reference found.
Location- Type of site/Geography	
No reference found.	No reference found.
Type of Service	
No reference found.	No reference found.
Supervision	
Supervision of an occupational therapy assistant provided by an occupational therapist must include monthly on-site contact with interim contact occurring by other means, including telephone, electronic mail, text messaging, and written communication. ***Source:******Kan. Admin. Regs. § 100-54-12***	No reference found.
Informed Consent	
No reference found.	No reference found.
Patient-Provider-Relationship/In-Person Exam Required	
No reference found.	No reference found.
Licensing	
No reference found.	No reference found.
Other	
No reference found.	No reference found.

**Table:**

Kentucky	
Occupational Therapy	Physical Therapy
Telehealth/Telemedicine/Telecommunications Definition	
Telehealth means the use of interactive audio, video, or other electronic media to deliver health care. It includes the use of electronic media for diagnosis, consultation, treatment, transfer of health or medical data, and continuing education. ***Source:******Ky. Rev. Stat. Ann. § 26:319A.300*** Telehealth occupational therapy means the therapeutic use of purposeful and meaningful occupations (goal-directed activities) to evaluate and treat individuals who have a disease or disorder, impairment, activity limitation, or participation restriction that interferes with their ability to function independently in daily life roles, and to promote health and wellness practiced between the occupational therapist or occupational therapist assistant and the patient that is provided using: An electronic communication technology; or Two (2) way, interactive, simultaneous audio and video. ***Source:******201 Ky. Admin. Regs. 28:235***	Telehealth means the use of interactive audio, video, or other electronic media to deliver health care. It includes the use of electronic media for diagnosis, consultation, treatment, transfer of health or medical data, and continuing education. ***Source:******Ky. Rev. Stat. Ann. § 26:327.200*** Telephysical therapy means physical therapy between the credential holder and the patient who are not at the same physical location using interactive, secure, synchronous audio, and synchronous videoconferencing technology. ***Source:******201 Ky. Admin. Regs. 22:001***
Modality	
Occupational therapists may use live video and audio. ***Source:******Ky. Rev. Stat. Ann. § 319A.300******&******201 Ky. Admin. Regs. 28:235***	Physical therapists may use live video and audio. ***Source:******Ky. Rev. Stat. Ann. § 26:327.200******&******201 Ky. Admin. Regs. 22:001***
Location- Type of site/Geography	
No reference found.	No reference found.
Type of Service	
Occupational therapists must limit the practice of telehealth occupational therapy to the area of competence in which proficiency has been gained through education, training, and experience. ***Source:******201 Ky. Admin. Regs. 28:235***	Telehealth may be used for diagnosis, consultation, treatment, transfer of health or medical data, and continuing education. ***Source:******Ky. Rev. Stat. Ann. § 26:327.200***
Supervision	
No reference found.	No reference found.
Informed Consent	
The treating occupational therapist must ensure in writing that: Informed consent of the patient, or another appropriate person with authority to make the health care treatment decision for the patient, is obtained before services are provided through telehealth; and That the confidentiality of the patient’s medical information is maintained as required by Kentucky law. At a minimum, confidentiality shall be maintained through appropriate processes, practices, and technology as designated by the board and that conform to applicable federal law. ***Source:******Ky. Rev. Stat. Ann. § 319A.300*** A credential holder using telehealth to deliver occupational therapy services shall, upon initial contact with the client: Make attempts to verify the identity of the client; Obtain alternative means of contacting the client other than electronically such as by the use of a telephone number or mailing address; Provide to the client alternative means of contacting the credential holder other than electronically such as by the use of a telephone number or mailing address; Provide contact methods of alternative communication the credential holder shall use for emergency purposes such as an emergency on call telephone number; Document if the client has the necessary knowledge and skills to benefit from the type of telehealth provided by the credential holder; Use secure communications with clients, including encrypted text messages via e-mail or secure Web sites, and not use personal identifying information in non-secure communications and; Inform the client in writing about: The limitations of using technology in the provision of telehealth occupational therapy services; Potential risks to confidentiality of information, or inadvertent access of protected health information, due to technology in the provision of telehealth occupational therapy services; Potential risks of disruption in the use of telehealth occupational therapy services; When and how the credential holder will respond to routine electronic messages; In what circumstances the credential holder will use alternative communications for emergency purposes; Who else may have access to client communications with the credential holder; How communications can be directed to a specific credential holder; How the credential holder stores electronic communications from the client; and How the credential holder may elect to discontinue the provision of services through telehealth. ***Source:******201 Ky. Admin. Regs. 28:235***	The treating physical therapist must ensure that: Informed consent of the patient, or another appropriate person with authority to make the health care treatment decision for the patient, is obtained before services are provided through telehealth; and That the confidentiality of the patient’s medical information is maintained as required by Kentucky law. At a minimum, confidentiality shall be maintained through appropriate processes, practices, and technology as designated by the board and that conform to applicable federal law. A credential holder using telehealth to deliver physical therapy services or who practices telephysical therapy shall, upon an initial contact with the patient: Verify the identity of the patient; Obtain alternative means of contacting the patient; Provide to the patient alternative means of contacting the credential holder; Provide contact methods of alternative communication the credential holder shall use for emergency purposes; Not use personal identifying information in non-secure communications; an Inform the patient and document acknowledgement of the risk and limitations of: The use of electronic communications in the provision of physical therapy; The potential breach of confidentiality, or inadvertent access, of protected health information using electronic communication in the provision of physical therapy; and The potential disruption of electronic communication in the use of telephysical therapy. ***Source:******201 Ky. Admin. Regs. 28:235***
Patient-Provider-Relationship/In-Person Exam Required	
No reference found.	No reference found.
Licensing	
Occupational therapists must be licensed in the location where the patient is domiciled. If the occupational therapist is located outside of Kentucky, must submit to the Kentucky Board of Occupational Therapy the following: The name, permanent address, address in Kentucky, email address, and telephone number where the therapist can be reached; The name, business address, and telephone number of the licensed occupational therapist with whom the therapist is associated; The dates on which the therapist intends to practice in Kentucky; and A copy of the current license from the state in which the therapist is licensed along with a statement from the licensing authority that the individual is in good standing. Or A letter of verification issued by the NBCOT stating that the therapist meets the current requirements for certification as a registered occupational therapist or a certified occupational therapy assistant and is in good standing. ***Source:******201 Ky. Admin. Regs. 28:235******&******201 Ky. Admin. Regs. 28:030***	Kentucky is part of the Physical Therapy Licensure Compact. Physical therapists licensed through the compact in other compact states may provide services in Kentucky. ***Source:******Ky. Rev. Stat. Ann. § 327.300***
Other	
A credential holder using telehealth to deliver occupational therapy services or who practices telehealth occupational therapy shall: Limit the practice of telehealth occupational therapy to the area of competence in which proficiency has been gained through education, training, and experience; Maintain current competency in the practice of telehealth occupational therapy through continuing education, consultation, or other procedures, in conformance with current standards of scientific and professional knowledge. Document the client’s presenting problem, purpose, or diagnosis; Follow the record keeping requirements; and Ensure the confidential communications obtained and stored electronically shall not be recovered and accessed by unauthorized persons when the credential holder disposes of electronic equipment and data. ***Source:******201 Ky. Admin. Regs. 28:235***	A credential holder using electronic communication to deliver physical therapy services or who practices telephysical therapy shall: Be responsible for determining and documenting that telehealth is appropriate in the provision of physical therapy; Limit the practice of telephysical therapy to the area of competence in which proficiency has been gained through education, training, and experience; Document which physical therapy services were provided by telephysical therapy; Follow the Standards for Documentation; and Ensure that confidential communications obtained and stored electronically shall not be recovered and accessed by unauthorized persons when the credential holder disposes of electronic equipment and data. ***Source:******201 Ky. Admin. Regs. 28:235***

**Table:**

Louisiana	
Occupational Therapy	Physical Therapy
Telehealth/Telemedicine/Telecommunications Definition	
No reference found.	No reference found.
Modality	
Occupational therapists and occupational therapy assistants may use live video and audio telecommunications during supervision and client conferences. This excludes the use of store-and-forward. ***Source:******La. Admin. Code tit. 46, § 4903***	No reference found.
Location- Type of site/Geography	
No reference found.	No reference found.
Type of Service	
Louisiana allows the use of telecommunication, except when required to be face-to-face, between a supervising occupational therapist and an occupational therapy assistant that allows for simultaneous discussion for: Client care conferences Supervision of occupational therapy assistants Louisiana does not allow for other modalities. ***Source:******La. Admin. Code tit. 46, § 4903******&******La. Admin. Code tit. 46, § 4925***	No reference found.
Supervision	
A telephone or telecommunications that allow for simultaneous discussion may be used to facilitate communication between a supervising occupational therapist and an occupational therapist assistant. ***Source:******La. Admin. Code tit. 46, § 4925***	No reference found.
Informed Consent	
No reference found.	No reference found.
Patient-Provider-Relationship/In-Person Exam Required	
Louisiana requires a supervising occupational therapist to have evaluated and/or treated a patient prior to holding a client care conference with an occupational therapy assistant. ***Source:******La. Admin. Code tit. 46, § 4903***	No reference found.
Licensing	
No reference found.	Louisiana is part of the Physical Therapy Licensure Compact. Physical therapists licensed through the compact may provide services in other compact states and in Louisiana. ***Source:******La. Stat. Ann. § 37:2425***
Other	
No reference found.	No reference found.

**Table:**

Maine	
Occupational Therapy	Physical Therapy
Telehealth/Telemedicine/Telecommunications Definition	
No reference found.	No reference found.
Modality	
No reference found.	No reference found.
Location- Type of site/Geography	
No reference found.	No reference found.
Type of Service	
No reference found.	No reference found.
Supervision	
No reference found.	No reference found.
Informed Consent	
No reference found.	No reference found.
Patient-Provider-Relationship/In-Person Exam Required	
No reference found.	No reference found.
Licensing	
No reference found.	No reference found.
Other	
No reference found.	No reference found.

**Table:**

Maryland	
Occupational Therapy	Physical Therapy
Telehealth/Telemedicine/Telecommunications Definition	
Telehealth is the application of evaluative, consultative, preventative, and therapeutic services delivered through telecommunication and information technologies. Telehealth services can be synchronous, delivered through interactive technologies in real time or asynchronous, using store-and-forward technologies. Occupational therapy practitioners can use telehealth as a mechanism to provide services at a location that is physically distant from the client, thus allowing for services to occur where the client lives, works, and plays, if that is needed or desired. ***Source:******Maryland Board of Occupational Therapy Practice Position Statement—Telehealth OT and OTA Authority to Use (2013).***	No reference found.
Modality	
Occupational therapists are permitted to use live video and store-and-forward telehealth. ***Source:******Maryland Board of Occupational Therapy Practice Position Statement—Telehealth OT and OTA Authority to Use (2013).***	No reference found.
Location- Type of site/Geography	
Provider and patient must both be located within Maryland. ***Source:******Maryland Board of Occupational Therapy Practice Position Statement—Telehealth OT and OTA Authority to Use (2013).***	No reference found.
Type of Service	
Telehealth may be used to provide evaluative, consultative, preventative, and therapeutic services. ***Source:******Maryland Board of Occupational Therapy Practice Position Statement—Telehealth OT and OTA Authority to Use (2013).***	No reference found.
Supervision	
No reference.	No reference found.
Informed Consent	
No reference.	No reference found.
Patient-Provider-Relationship/In-Person Exam Required	
No reference.	No reference found.
Licensing	
Occupational therapy personnel must be licensed in Maryland prior to providing services via telehealth. Practice delivered via telehealth must be in accordance with Maryland statute and regulations. ***Source:******Maryland Board of Occupational Therapy Practice Position Statement—Telehealth OT and OTA Authority to Use (2013).***	No reference found.
Other	
No reference found.	No reference found.

**Table:**

Massachusetts	
Occupational Therapy	Physical Therapy
Telehealth/Telemedicine/Telecommunications Definition	
No reference found.	No reference found.
Modality	
No reference found.	No reference found.
Location- Type of site/Geography	
No reference found.	No reference found.
Type of Service	
No reference found.	No reference found.
Supervision	
No reference found.	No reference found.
Informed Consent	
No reference found.	No reference found.
Patient-Provider-Relationship/In-Person Exam Required	
No reference found.	No reference found.
Licensing	
No reference found.	No reference found.
Other	
No reference found.	No reference found.

**Table:**

Michigan	
Occupational Therapy	Physical Therapy
Telehealth/Telemedicine/Telecommunications Definition	
No reference found.	No reference found.
Modality	
Telecommunications, which would include live video and audio and telephone, may be used for some supervision. ***Source:******Mich. Admin. Code r. 338.1229***	Telecommunications, which would include live video and audio and telephone, may be used for some supervision. ***Source:******Mich. Admin. Code r. 338.7138***
Location- Type of site/Geography	
No reference found.	No reference found.
Type of Service	
No reference found.	No reference found.
Supervision	
Occupational therapists are permitted to use telecommunications or other electronic devices to provide general supervision to occupational therapy assistants. ***Source:******Mich. Admin. Code r. 338.1229***	Physical therapists are permitted to use telecommunications or other electronic devices to provide general supervision to physical therapy assistants. ***Source:******Mich. Admin. Code r. 338.7138***
Informed Consent	
No reference found.	No reference found.
Patient-Provider-Relationship/In-Person Exam Required	
No reference found.	No reference found.
Licensing	
No reference found.	No reference found.
Other	
No reference found.	No reference found.

**Table:**

Minnesota	
Occupational Therapy	Physical Therapy
Telehealth/Telemedicine/Telecommunications Definition	
No reference found.	No reference found.
Modality	
No reference found.	Physical therapists may use live video and audio telecommunications during supervision of physical therapy assistants. ***Source:******Minn. Stat. § 148.706***
Location- Type of site/Geography	
No reference found.	No reference found.
Type of Service	
No reference found.	No reference found.
Supervision	
No reference found.	Telecommunications, except within a facility, does not meet the requirements of on-site supervision when provided to a student physical therapist. ***Source:******Minn. Stat. § 148.65*** A supervising physical therapist is not required to be on-site while providing supervision to a physical therapy assistant, but must be easily available via telecommunications. ***Source:******Minn. Stat. § 148.706***
Informed Consent	
No reference found.	No reference found.
Patient-Provider-Relationship/In-Person Exam Required	
No reference found.	No reference found.
Licensing	
No reference found.	No reference found.
Other	
No reference found.	No reference found.

**Table:**

Mississippi	
Occupational Therapy	Physical Therapy
Telehealth/Telemedicine/Telecommunications Definition	
No reference found.	No reference found.
Modality	
Telecommunications, which would include live video and audio and telephone, may be used for some supervision. ***Source:******Miss. Code R. § 15-019-008***	Telecommunications, which would include live video and audio and telephone, may be used for some supervision. ***Source:******Miss. Code R. § 300-031-3103***
Location- Type of site/Geography	
No reference found.	No reference found.
Type of Service	
No reference found.	Telehealth is an appropriate model of service delivery when it is provided in a manner consistent with the standards of practice, ethical principles, rules and regulations for Mississippi physical therapy practitioners. ***Source:******Miss. Code R. § 300-031-3103***
Supervision	
A supervising occupational therapist must be accessible via telecommunications to the occupational therapy assistant on a daily basis. ***Source:******Miss. Code R. § 15-019-008***	A supervising physical therapist may be available for direct and/or on-site supervision to a physical therapist assistant via telecommunications when not on the premises. ***Source:******Miss. Code R. § 300-031-3103***
Informed Consent	
No reference found.	No reference found.
Patient-Provider-Relationship/In-Person Exam Required	
No reference found.	No reference found.
Licensing	
No reference found.	Mississippi is part of the Physical Therapy Licensure Compact. Physical therapists licensed through the compact in other compact states may provide services in Mississippi. ***Source:******Miss. Code Ann. § 73-23-101***
Other	
No reference found.	No reference found.

**Table:**

Missouri	
Occupational Therapy	Physical Therapy
Telehealth/Telemedicine/Telecommunications Definition	
No reference found.	No reference found.
Modality	
No reference found.	Telecommunications, which would include live video and audio and telephone, may be used for some supervision. ***Source:******Mo. Code Regs. Tit.20, § 2150-3.090***
Location- Type of site/Geography	
No reference found.	No reference found.
Type of Service	
No reference found.	No reference found.
Supervision	
No reference found.	A supervising physical therapist must be accessible via telecommunications at all times by a physical therapist assistant. ***Source:******Mo. Code Regs. Tit.20, § 2150-3.090***
Informed Consent	
No reference found.	No reference found.
Patient-Provider-Relationship/In-Person Exam Required	
No reference found.	No reference found.
Licensing	
No reference found.	Missouri is part of the Physical Therapy Licensure Compact. Physical therapists licensed through the compact in other compact states may provide services in Missouri. ***Source:******Mo. Rev. Stat. § 334.1200***
Other	
No reference found.	No reference found.

**Table:**

Montana	
Occupational Therapy	Physical Therapy
Telehealth/Telemedicine/Telecommunications Definition	
No reference found.	“Telemedicine” means the use of interactive audio, video, or other telecommunications technology that is: Used by a health care provider or health care facility to deliver health care services at a site other than the site where the patient is located; and Delivered over a secure connection that complies with the requirements of the Health Insurance Portability and Accountability Act of 1996, 42 U.S.C. 1320d, et seq. Telehealth includes the use of electronic media for consultation relating to the health care diagnosis or treatment of a patient in real time or through the use of store-and-forward technology. ***Source:******Mont. Code Ann. § 33-22-138***
Modality	
Telephone and electronic communication may be used to provide some supervision. ***Source:******Mont. Admin. R. 24.165.502***	Telehealth includes live video and store-and-forward. Audio-only telephone, e-mail, and facsimile transmissions are not included under telehealth. “Store-and-forward” technology means electronic information, imaging, and communication that is transferred, recorded, or otherwise stored in order to be reviewed at a later date by a health care provider or health care facility at a distant site without the patient present in real time. The term includes interactive audio, video, and data communication. ***Source:******Mont. Code Ann. § 33-22-138***
Location- Type of site/Geography	
No reference found.	No reference found.
Type of Service	
No reference found.	Telehealth may be used to provide consultation relating to a health care diagnosis or treatment or to provide health care services. ***Source:******Mont. Code Ann. § 33-22-138***
Supervision	
Occupational therapists and certified occupational therapy assistants are permitted to use telephonic, electronic, or other written communication to meet requirements for interim routine and general supervision. ***Source:******Mont. Admin. R. 24.165.502***	No reference found.
Informed Consent	
No reference found.	No reference found.
Patient-Provider-Relationship/In-Person Exam Required	
No reference found.	No reference found.
Licensing	
No reference found.	Montana is part of the Physical Therapy Licensure Compact. Physical therapists licensed through the compact may provide services in other compact states and in Montana. ***Source:******Mont. Code Ann. § 37-11-316***
Other	
No reference found.	No reference found.

**Table:**

Nebraska	
Occupational Therapy	Physical Therapy
Telehealth/Telemedicine/Telecommunications Definition	
No reference found.	Telecommunications means a land phone-line, cellular phone service, pager, video teleconference or any similar teleconferencing that will allow immediate response time. Facsimile and electronic mail are not defined as telecommunications due to inadequate response time. ***Source:******172 Neb. Admin. Code, ch. 137, § 002***
Modality	
No reference found.	Live video, cellular phone, land phone-line, and pager may be used for some supervision. Facsimile and electronic mail are not considered telecommunications. ***Source:******172 Neb. Admin. Code, ch. 137, § 002***
Location- Type of site/Geography	
No reference found.	No reference found.
Type of Service	
No reference found.	No reference found.
Supervision	
No reference found.	A physical therapist may provide general supervision to a physical therapist assistant either in-person or via telecommunications. ***Source:******Neb. Rev. Stat. § 38-2908******&******172 Neb. Admin. Code, ch. 137, § 002***
Informed Consent	
No reference found.	No reference found.
Patient-Provider-Relationship/In-Person Exam Required	
No reference found.	No reference found.
Licensing	
No reference found.	Nebraska is part of the Physical Therapy Licensure Compact. Physical therapists licensed through the compact may provide services in other compact states and in Nebraska. ***Source:******Neb. Rev. Stat. § 38-4001***
Other	
No reference found.	General supervision is defined as being either on-site or by means of telecommunications. Use of telecommunications for general supervision is required to be documented in a patient’s medical records by the supervising physical therapist. Direct supervision requires face-to-face interaction and cannot be provided by telecommunications. ***Source:******172 Neb. Admin. Code, ch. 137, § 002***

**Table:**

Nevada	
Occupational Therapy	Physical Therapy
Telehealth/Telemedicine/Telecommunications Definition	
“Telehealth” means the delivery of services from a provider of health care to a patient at a different location through the use of information and audio-visual communication technology, not including standard telephone, facsimile or electronic mail. ***Source:******Nev. Rev. Stat. § 629.515***	“Telehealth” means the delivery of services from a provider of health care to a patient at a different location through the use of information and audio-visual communication technology, not including standard telephone, facsimile or electronic mail. ***Source:******Nev. Rev. Stat. § 629.515***
Modality	
Audio-visual communication technology is included in telehealth definition. Telehealth does not include standard telephone, facsimile or electronic mail. ***Source:******Nev. Rev. Stat. § 629.515***	Audio-visual communication technology is included in telehealth definition. Telehealth does not include standard telephone, facsimile or electronic mail. ***Source:******Nev. Rev. Stat. § 629.515***
Location- Type of site/Geography	
“Distant site” means the location of the site where a telehealth provider of health care is providing telehealth services to a patient located at an originating site. “Originating site” means the location of the site where a patient is receiving telehealth services from a provider of health care located at a distant site. ***Source:******Nev. Rev. Stat. § 629.515***	“Distant site” means the location of the site where a telehealth provider of health care is providing telehealth services to a patient located at an originating site. “Originating site” means the location of the site where a patient is receiving telehealth services from a provider of health care located at a distant site. ***Source:******Nev. Rev. Stat. § 629.515***
Type of Service	
No reference found.	No reference found.
Supervision	
No reference found.	No reference found.
Informed Consent	
No reference found.	No reference found.
Patient-Provider-Relationship/In-Person Exam Required	
No reference found.	No reference found.
Licensing	
Occupational therapists must be licensed in Nevada prior to providing telehealth services to patients, unless they are providing services within the scope of their employment or pursuant to a contract entered into with an urban Indian organization. ***Source:******Nev. Rev. Stat. § 629.515***	Physical therapists must be licensed in Nevada prior to providing telehealth services to patients, unless they are providing services within the scope of their employment or pursuant to a contract entered into with an urban Indian organization. ***Source:******Nev. Rev. Stat. § 629.515***
Other	
	The Nevada Board of Physical Therapy Examiners states that telehealth falls within the regular scope of practice for physical therapy. ***Source:******Nevada Board of Physical Therapy Examiners Draft Board Meeting Minutes February 23, 2017***

**Table:**

New Hampshire	
Occupational Therapy	Physical Therapy
Telehealth/Telemedicine/Telecommunications Definition	
No reference found.	No reference found.
Modality	
Telephone and electronic communication may be used to provide some supervision. ***Source:******N.H. Code Admin. R. Occ. 301.05***	Telecommunications may be used to provide some ssupervision. ***Source:******N.H. Rev. Stat. Ann. § 328-A:2***
Location- Type of site/Geography	
No reference found.	No reference found.
Type of Service	
No reference found.	“Consultation by means of telecommunication” means that a physical therapist renders professional or expert opinion or advice to another physical therapist or health care provider via telecommunications or computer technology from a distant location. It includes the transfer or exchange of educational or related information by means of audio, video, or data communications. ***Source:******N.H. Rev. Stat. Ann. § 328-A:2***
Supervision	
New Hampshire allows indirect supervision, or any other form of supervision that is not direct supervision, to be conducted via telephone conversations, electronic correspondence. ***Source:******N.H. Code Admin. R. Occ. 301.05***	General supervision means that a supervising physical therapist is not required to be on-site but must be available through telecommunications. Telecommunications may not be used for direct or direct personal supervision, which require a physical therapist to be physically present. ***Source:******N.H. Rev. Stat. Ann. § 328-A:2***
Informed Consent	
No reference found.	No reference found.
Patient-Provider-Relationship/In-Person Exam Required	
No reference found.	No reference found.
Licensing	
No reference found.	New Hampshire is part of the Physical Therapy Licensure Compact. Physical therapists licensed through the compact may provide services in other compact states and in New Hampshire. ***Source:******N.H. Rev. Stat. Ann. § 328-A:5-a***
Other	
No reference found.	No reference found.

**Table:**

New Jersey	
Occupational Therapy	Physical Therapy
Telehealth/Telemedicine/Telecommunications Definition	
“Telehealth” means the use of information and communications technologies, including telephones, remote patient monitoring devices, or other electronic means, to support clinical health care, provider consultation, patient and professional health-related education, public health, health administration, and other services in accordance with New Jersey Law. “Telemedicine” means the delivery of a health care service using electronic communications, information technology, or other electronic or technological means to bridge the gap between a health care provider who is located at a distant site and a patient who is located at an originating site, either with or without the assistance of an intervening health care provider, and in accordance with New Jersey Law. “Telemedicine” does not include the use, in isolation, of audio-only telephone conversation, electronic mail, instant messaging, phone text, or facsimile transmission. ***Source:******N.J. Rev. Stat. § 45:1-61***	“Telehealth” means the use of information and communications technologies, including telephones, remote patient monitoring devices, or other electronic means, to support clinical health care, provider consultation, patient and professional health-related education, public health, health administration, and other services in accordance with New Jersey Law. “Telemedicine” means the delivery of a health care service using electronic communications, information technology, or other electronic or technological means to bridge the gap between a health care provider who is located at a distant site and a patient who is located at an originating site, either with or without the assistance of an intervening health care provider, and in accordance with New Jersey Law. “Telemedicine” does not include the use, in isolation, of audio-only telephone conversation, electronic mail, instant messaging, phone text, or facsimile transmission. ***Source:******N.J. Rev. Stat. § 45:1-61***
Modality	
Occupational therapists may use communications technology, telephones, remote patient monitoring, and other electronic communication to supplement services. ***Source:******N.J. Rev. Stat. § 45:1-61*** When providing health care services via telemedicine, an occupational therapist may only use synchronous telehealth. An occupational therapist is permitted to use asynchronous store-and-forward technology if they have reviewed the patient’s medical history and believe it is possible to provide the same level of care. ***Source:******N.J. Rev. Stat. § 45:1-62***	Physical therapists may use communications technology, telephones, remote patient monitoring, and other electronic communication to supplement services. ***Source:******N.J. Rev. Stat. § 45:1-61*** When providing health care services via telemedicine, a physical therapist may only use synchronous telehealth. A physical therapist is permitted to use asynchronous store-and-forward technology if they have reviewed the patient’s medical history and believe it is possible to provide the same level of care. ***Source:******N.J. Rev. Stat. § 45:1-62***
Location- Type of site/Geography	
“Distant site” means a site at which a health care provider, acting within the scope of a valid license or certification issued pursuant to Title 45 of the Revised Statutes, is located while providing health care services by means of telemedicine or telehealth. “Originating site” means a site at which a patient is located at the time that health care services are provided to the patient by means of telemedicine or telehealth. ***Source:******N.J. Rev. Stat. § 45:1-61***	“Distant site” means a site at which a health care provider, acting within the scope of a valid license or certification issued pursuant to Title 45 of the Revised Statutes, is located while providing health care services by means of telemedicine or telehealth. “Originating site” means a site at which a patient is located at the time that health care services are provided to the patient by means of telemedicine or telehealth. ***Source:******N.J. Rev. Stat. § 45:1-61***
Type of Service	
Occupational therapists may use telehealth or telemedicine for any service within their scope of practice. Occupational therapists may engage in remote evaluation of a patient at the request of another occupational therapist who has established a prior provider-patient relationship with the patient. ***Source:******N.J. Rev. Stat. § 45:1-61***	Physical therapists may use telehealth or telemedicine for any service within their scope of practice. Physical therapists may engage in remote evaluation of a patient at the request of another physical therapist who has established a prior provider-patient relationship with the patient. ***Source:******N.J. Rev. Stat. § 45:1-61***
Supervision	
No reference found.	No reference found.
Informed Consent	
Patient consent is required prior to forwarding the patient’s health record to the patient’s primary care provider after a service has been provide via telehealth or telemedicine. ***Source:******N.J. Rev. Stat. § 45:1-62***	Patient consent is required prior to forwarding the patient’s health record to the patient’s primary care provider after a service has been provide via telehealth or telemedicine. ***Source:******N.J. Rev. Stat. § 45:1-62***
Patient-Provider-Relationship/In-Person Exam Required	
An occupational therapist must have an established provider-patient relationship with a patient prior to providing remote services via telemedicine. ***Source:******N.J. Rev. Stat. § 45:1-62***	A physical therapist must have an established provider-patient relationship with a patient prior to providing remote services via telemedicine. ***Source:******N.J. Rev. Stat. § 45:1-62***
Licensing	
Occupational therapists must be licensed in New Jersey if providing telehealth services to patients located in the state. ***Source:******N.J. Rev. Stat. § 45:1-62***	New Jersey is part of the Physical Therapy Licensure Compact. Physical therapists licensed through the compact in other compact states may provide services in New Jersey. ***Source:******N.J. Rev. Stat. § 45:9-37.34h***
Other	
An occupational therapist must provide their identity, professional credentials, and contact information during and after services provided via telehealth or telemedicine. ***Source:******N.J. Rev. Stat. § 45:1-62***	A physical therapist must provide their identity, professional credentials, and contact information during and after services provided via telehealth or telemedicine. ***Source:******N.J. Rev. Stat. § 45:1-62***

**Table:**

New Mexico	
Occupational Therapy	Physical Therapy
Telehealth/Telemedicine/Telecommunications Definition	
“Telehealth” means the use of electronic information, imaging and communication technologies, including interactive audio, video, data communications as well as store-and-forward technologies, to provide and support health care delivery, diagnosis, consultation, treatment, transfer of medical data and education. ***Source:******N.M. Stat. § 24-25-3***	“Telehealth” means the use of electronic information, imaging and communication technologies, including interactive audio, video, data communications as well as store-and-forward technologies, to provide and support health care delivery, diagnosis, consultation, treatment, transfer of medical data and education. ***Source:******N.M. Stat. § 24-25-3***
Modality	
Live video and audio, as well as store-and-forward, may be used to deliver services. ***Source:******N.M. Stat. § 24-25-3*** Live video, electronic exchanges, telephone, and other secure telecommunication technology may be used for some supervision. ***Source:******N.M. Code R. § 16.15.3.8***	Live video and audio, as well as store-and-forward, may be used to deliver services. ***Source:******N.M. Stat. §24-25-3*** Phone, electronic mail or cellular phone may be used for some supervision. ***Source:******N.M. Code R. § 16.20.6.8***
Location- Type of site/Geography	
Originating site includes all of the following: A licensed inpatient center; An ambulatory surgical or treatment center; A skilled nursing center; A residential treatment center; A home health agency; A diagnostic laboratory or imaging center; An assisted living center; A school-based health program; A mobile clinic; A mental health clinic; A rehabilitation or other therapeutic health setting; A patient’s residence; A federally qualified health center; or A community health center ***Source:******N.M. Stat. § 24-25-3***	Originating site includes all of the following: A licensed inpatient center; An ambulatory surgical or treatment center; A skilled nursing center; A residential treatment center; A home health agency; A diagnostic laboratory or imaging center; An assisted living center; A school-based health program; A mobile clinic; A mental health clinic; A rehabilitation or other therapeutic health setting; A patient’s residence; A federally qualified health center; or A community health center ***Source:******N.M. Stat. § 24-25-3***
Type of Service	
Telehealth may be used to provide and support health care delivery, diagnosis, consultation, treatment, transfer of medical data and education. ***Source:******N.M. Stat. § 24-25-3*** New Mexico does not alter the scope of practice or authorize the delivery of health care in a setting or manner not otherwise authorized by law when care is provided via telehealth. ***Source:******N.M. Stat. § 24-25-5***	Telehealth may be used to provide and support health care delivery, diagnosis, consultation, treatment, transfer of medical data and education. ***Source:******N.M. Stat. § 24-25-3*** New Mexico does not alter the scope of practice or authorize the delivery of health care in a setting or manner not otherwise authorized by law when care is provided via telehealth. ***Source:******N.M. Stat. § 24-25-5***
Supervision	
Occupational therapists may use video teleconferencing to provide supervision to occupational therapy assistants, that requires direct, face-to-face contact. When providing supervision that involves indirect contact, an occupational therapist may use: Phone conversations Electronic exchanges Other methods using secure telecommunication technology All methods of communication must be compliant with confidentiality requirements of government agencies, facilities, employers, or other appropriate bodies. ***Source:******N.M. Code R. § 16.15.3.8***	For direction and supervision, a physical therapist must be readily available to the physical therapist assistant via phone, electronic mail or cellular phone whenever they leave the facility OR the referring physical therapist must appoint a stand in physical therapist to supervise. Other requirements apply. ***Source:******N.M. Code R. § 16.20.6.8***
Informed Consent	
No reference found.	No reference found.
Patient-Provider-Relationship/In-Person Exam Required	
No reference found.	No reference found.
Licensing	
Occupational therapists must be licensed in New Mexico if providing telehealth services to a patient located within the state. ***Source:******N.M. Stat. § 24-25-3***	Physical therapists must be licensed in New Mexico if providing telehealth services to a patient located within the state. ***Source:******N.M. Stat. § 24-25-3***
Other	
Telehealth is recognized and encouraged in New Mexico. Occupational therapists must comply with all applicable federal and state guidelines regarding security, confidentiality and privacy protections for health care information. ***Source:******N.M. Stat. § 24-25-4*** Occupational therapists and occupational therapy assistants are required to document a supervision plan and supervision contacts, which must include the method of communication. ***Source:******N.M. Code R. § 16.15.3.8***	Telehealth is recognized and encouraged in New Mexico. Physical therapists must comply with all applicable federal and state guidelines regarding security, confidentiality and privacy protections for health care information. ***Source:******N.M. Stat. § 24-25-4***

**Table:**

New York	
Occupational Therapy	Physical Therapy
Telehealth/Telemedicine/Telecommunications Definition	
“Telehealth” means the use of electronic information and communication technologies by telehealth providers to deliver health care services, which shall include the assessment, diagnosis, consultation, treatment, education, care management and/or self-management of a patient. Telehealth shall not include delivery of health care services by means of audio-only telephone communication, facsimile machines, or electronic messaging alone, though use of these technologies is not precluded if used in conjunction with telemedicine, store and forward technology, or remote patient monitoring. For purposes of this section, telehealth shall be limited to telemedicine, store-and-forward technology, and remote patient monitoring. This subdivision shall not preclude the delivery of health care services by means of “home telehealth.” “Telemedicine” means the use of synchronous, two-way electronic audio visual communications to deliver clinical health care services, which shall include the assessment, diagnosis, and treatment of a patient, while such patient is at the originating site and a telehealth provider is at a distant site. ***Source:******N.Y. P.B.H. Law § 2999-CC***	“Telehealth” means the use of electronic information and communication technologies by telehealth providers to deliver health care services, which shall include the assessment, diagnosis, consultation, treatment, education, care management and/or self-management of a patient. Telehealth shall not include delivery of health care services by means of audio-only telephone communication, facsimile machines, or electronic messaging alone, though use of these technologies is not precluded if used in conjunction with telemedicine, store and forward technology, or remote patient monitoring. For purposes of this section, telehealth shall be limited to telemedicine, store-and-forward technology, and remote patient monitoring. This subdivision shall not preclude the delivery of health care services by means of “home telehealth.” “Telemedicine” means the use of synchronous, two-way electronic audio visual communications to deliver clinical health care services, which shall include the assessment, diagnosis, and treatment of a patient, while such patient is at the originating site and a telehealth provider is at a distant site. ***Source:******N.Y. P.B.H. Law § 2999-CC***
Modality	
Occupational therapists may use synchronous, two-way electronic audio-visual communications, store-and-forward technology, and remote patient monitoring. Audio-only telephone, facsimile machine, or electronic messaging alone are not considered telehealth. “Store and forward technology” means the asynchronous, electronic transmission of a patient’s health information in the form of patient-specific digital images and/or pre-recorded videos from a provider at an originating site to a telehealth provider at a distant site. “Remote patient monitoring” means the use of synchronous or asynchronous electronic information and communication technologies to collect personal health information and medical data from a patient at an originating site that is transmitted to a telehealth provider at a distant site for use in the treatment and management of medical conditions that require frequent monitoring. Such technologies may include additional interaction triggered by previous transmissions, such as interactive queries conducted through communication technologies or by telephone. Such conditions shall include, but not be limited to, congestive heart failure, diabetes, chronic obstructive pulmonary disease, wound care, polypharmacy, mental or behavioral problems, and technology-dependent care such as continuous oxygen, ventilator care, total parenteral nutrition or enteral feeding. Remote patient monitoring may only be ordered by a physician, nurse practitioner or nurse midwife, with which the patient has a substantial and ongoing relationship. ***Source:******N.Y. P.B.H. Law § 2999-CC***	Physical therapists may use synchronous, two-way electronic audio visual communications, store-and-forward technology, and remote patient monitoring. Audio-only telephone, facsimile machine, or electronic messaging alone are not considered telehealth. “Store and forward technology” means the asynchronous, electronic transmission of a patient’s health information in the form of patient-specific digital images and/or pre-recorded videos from a provider at an originating site to a telehealth provider at a distant site. “Remote patient monitoring” means the use of synchronous or asynchronous electronic information and communication technologies to collect personal health information and medical data from a patient at an originating site that is transmitted to a telehealth provider at a distant site for use in the treatment and management of medical conditions that require frequent monitoring. Such technologies may include additional interaction triggered by previous transmissions, such as interactive queries conducted through communication technologies or by telephone. Such conditions shall include, but not be limited to, congestive heart failure, diabetes, chronic obstructive pulmonary disease, wound care, polypharmacy, mental or behavioral problems, and technology-dependent care such as continuous oxygen, ventilator care, total parenteral nutrition or enteral feeding. Remote patient monitoring may only be ordered by a physician, nurse practitioner or nurse midwife, with which the patient has a substantial and ongoing relationship. ***Source:******N.Y. P.B.H. Law § 2999-CC***
Location- Type of site/Geography	
“Distant site” means a site at which a telehealth provider is located while delivering health care services by means of telehealth. “Originating site” means a site at which a patient is located at the time health care services are delivered to him or her by means of telehealth. Originating sites shall be limited to a: Hospitals and General Hospitals; Nursing homes; Residential Health Care Facilities; Out-patient lodge; Midwifery Birth Center; Hospice or Hospice Residence; Facilities where services for the mentally disabled are provided; Certified and non-certified day and residential programs funded or operated by the office for people with developmental disabilities; Private physician’s or dentist’s offices located within the state of New York; Any type of adult care facility licensed under title two of article seven of the social services law; Public, private and charter elementary and secondary schools, school age child care programs, and child day care centers within the state of New York; and The patient’s place of residence located within the state of New York or other temporary location located within or outside the state of New York. ***Source:******N.Y. P.B.H. Law § 2999-CC***	“Distant site” means a site at which a telehealth provider is located while delivering health care services by means of telehealth. “Originating site” means a site at which a patient is located at the time health care services are delivered to him or her by means of telehealth. Originating sites shall be limited to a: Hospitals and General Hospitals; Nursing homes; Residential Health Care Facilities; Out-patient lodge; Midwifery Birth Center; Hospice or Hospice Residence; Facilities where services for the mentally disabled are provided; Certified and non-certified day and residential programs funded or operated by the office for people with developmental disabilities; Private physician’s or dentist’s offices located within the state of New York; Any type of adult care facility licensed under title two of article seven of the social services law; Public, private and charter elementary and secondary schools, school age child care programs, and child day care centers within the state of New York; and The patient’s place of residence located within the state of New York or other temporary location located within or outside the state of New York. ***Source:******N.Y. P.B.H. Law § 2999-CC***
Type of Service	
Telehealth includes the assessment, diagnosis, consultation, treatment, education, care management and/or self-management of a patient. ***Source:******N.Y. P.B.H. Law § 2999-CC***	Telehealth includes the assessment, diagnosis, consultation, treatment, education, care management and/or self-management of a patient. ***Source:******N.Y. P.B.H. Law § 2999-CC***
Supervision	
No reference found.	No reference found.
Informed Consent	
No reference found.	No reference found.
Patient-Provider-Relationship/In-Person Exam Required	
No reference found.	No reference found.
Licensing	
Occupational therapists must be licensed in New York if providing telehealth services to a patient located within the state. ***Source:******N.Y. P.B.H. Law § 2999-CC***	Physical therapists must be licensed in New York if providing telehealth services to a patient located within the state. ***Source:******N.Y. P.B.H. Law § 2999-CC***
Other	
Occupational therapists are included in the definition of a telehealth provider. ***Source:******N.Y. P.B.H. Law § 2999-CC***	Physical therapists are included in the definition of a telehealth provider. ***Source:******N.Y. P.B.H. Law § 2999-CC*** Physical therapy telepractice is subject to all practice and ethical considerations governing physical therapy practice in New York State. Physical therapists should consider the particular impact of telepractice on dimensions of physical therapy practice, including, but not limited to: Awareness and assessment of unobservable behavior; Confidentiality and privacy of clients and their transmissions; Access issues such as distribution of computers and familiarity with technology; Temporal factors such as simultaneous communication, time between responses, and formalized “sessions”; and Development of technological proficiencies and on-line culture/language. ***Source:******N.Y. E.D.N. Law § 6509(2)***

**Table:**

North Carolina	
Occupational Therapy	Physical Therapy
Telehealth/Telemedicine/Telecommunications Definition	
No information found.	No information found.
Modality	
Synchronous or asynchronous video teleconferencing may be used to provide some supervision. ***Source:******NC Board of Occupational Therapy Telehealth Guidance (Aug. 2018)***	Telecommunications may be used to provide some supervision. ***Source:******21 N.C. Admin. Code 48C .0102***
Location- Type of site/Geography	
No information found.	No information found.
Type of Service	
An occupational therapy practitioner may deliver evaluation, treatment, and consultation through telecommunication and information technologies. ***Source:******NC Board of Occupational Therapy Telehealth Guidance (Aug. 2018)***	No information found.
Supervision	
Occupational therapy practitioners may provide general supervision via videoconferencing or other telecommunication technology. The observation may be synchronous or asynchronous. However, direct supervision does not include the use of video conferencing. ***Source:******21 N.C. Admin. Code 38 .0103(21)*** An occupational therapy practitioner may provide supervision requiring direct contact via video teleconferencing. ***Source:******NC Board of Occupational Therapy Telehealth Guidance (Aug. 2018)***	Physical therapists are required to be immediately available either in person or via telecommunications to physical therapist assistants supervising physical therapy aides or students engaging in patient care. ***Source:******21 N.C. Admin. Code 48C .0102***
Informed Consent	
No information found.	No information found.
Patient-Provider-Relationship/In-Person Exam Required	
No information found.	No information found.
Licensing	
An occupational therapist must be licensed in North Carolina to provide services to a client who is in North Carolina. An occupational therapist licensed in North Carolina who is located in another state may provide services to clients located in North Carolina. An occupational therapist located in North Carolina who does not provide services to clients in North Carolina does not need to be licensed in North Carolina, however if the occupational therapist provides services to clients outside of North Carolina, they are required to follow the laws and regulations of the state where the client is receiving services. ***Source:******NC Board of Occupational Therapy Telehealth Guidance (Aug. 2018)***	North Carolina is part of the Physical Therapy Licensure Compact. Physical therapists licensed through the compact in other compact states may provide services in North Carolina. ***Source:******N.C. Gen. Stat. § 90-270.120-131******&******NC HB 57 (2017)***
Other	
No information found.	No information found.

**Table:**

North Dakota	
Occupational Therapy	Physical Therapy
Telehealth/Telemedicine/Telecommunications Definition	
Telemedicine means the practice of medicine by a practitioner, other than a pharmacist, who is at a location remote from the patient, and is communicating with the patient, or health care professional who is treating the patient, using a telecommunications system. Telehealth is a service delivery model that allows an occupational therapy practitioner to deliver evaluation, treatment, and consultation through telecommunication and information technologies overcoming distance, transportation expenses, and patient access barriers. ***Source:******ND Board of Occupational Therapy Practice Practice-Related Information (Aug. 2018).***	“Telehealth” is the use of electronic communications to provide and deliver a host of health related information and healthcare services, including, but not limited to physical therapy related information and services, over large and small distance. Telehealth encompasses a variety of healthcare and health promotion activities, including, but not limited to, education, advice, reminders, interventions, and monitoring of interventions. ***Source:******N.D. Admin. Code 61.5-01-02***
Modality	
Telehealth includes the use of telecommunication and information technologies, which would include live video and audio and telephone. ***Source:******ND Board of Occupational Therapy Practice Practice-Related Information (Aug. 2018).***	Telehealth includes the use of audio, video, or data communications. ***Source:******N.D. Admin. Code 61.5-01-02***
Location- Type of site/Geography	
The location of the patient at the time of the patient encounter determines the location of the service. Occupational therapy practitioners are required to be licensed in North Dakota if they are providing occupational therapy services to a client who is in North Dakota. If the therapist/assistant is connecting with a patient in another State at the time of the patient encounter, the therapist must be licensed in that state. ***Source:******ND Board of Occupational Therapy Practice Practice-Related Information (Aug. 2018).***	Physical therapists licensed in another US jurisdiction are exempt from North Dakota physical therapy licensure requirements if providing consultation by means of telecommunication to a physical therapist who is licensed in North Dakota. ***Source:******N.D. Admin. Code 61.5-01-02***
Type of Service	
Occupational therapists may use telehealth for evaluation, treatment, and consultation with a patient or other provider. ***Source:******ND Board of Occupational Therapy Practice Practice-Related Information (Aug. 2018).*** Occupational therapy services are provided for habilitation, rehabilitation, and the promotion of health and wellness, including methods delivered via telerehabilitation to those who have or are at risk for developing an illness, injury, disease, disorder, condition, impairment, disability, activity limitation, or participation restriction. ***Source: ND Admin. Code 55.5-03-01-03.***	A physical therapist may provide services that are legally or professionally authorized via telehealth. ***Source:******N.D. Admin. Code 61.5-01-02*** “Consultation by telecommunication” means that a physical therapist renders professional or expert opinion or advice to another physical therapist or health care provider via telecommunications or computer technology from a distant location. It includes the transfer or exchange of educational or related information by means of audio, video, or data communications. The physical therapist may use telehealth technology as a vehicle for providing only services that are legally or professionally authorized. All records used or resulting from a consultation by means of telecommunications are part of a patient’s record and are subject to applicable confidentiality requirements. ***Source:******N.D. Admin. Code 61.5-01-02***
Supervision	
Direct supervision means face-to-face contact including videoconferencing. Indirect supervision means other than face-to-face contact, including telephonic and electronic communication, and other methods using secure telecommunication technology. An occupational therapy assistant must be directly supervised and indirectly supervised as necessary. ***Source:******N.D. Admin Code 55.5-02-03-01.1*** An occupational therapy practitioner may provide occupational therapy personnel supervision requiring direct supervision and indirect supervision through electronic medical record technology and video teleconferencing. The practitioner will be responsible for the appropriate use of teleconferencing mediums in the supervision of services and maintain the privacy standards in all patient related interactions. ***Source:******ND Board of Occupational Therapy Practice Practice-Related Information (Aug. 2018).***	A physical therapist may not provide direct supervision via telecommunications. ***Source:******N.D. Admin. Code 61.5-01-02***
Informed Consent	
No reference found.	The patient’s written or verbal consent must be obtained and documented prior to a consultation by means of telecommunication. ***Source:******N.D. Admin. Code 61.5-01-02***
Patient-Provider-Relationship/In-Person Exam Required	
No reference found.	No reference found.
Licensing	
No reference found.	North Dakota is part of the Physical Therapy Licensure Compact. Physical therapists licensed through the compact may provide services in other compact states and in North Dakota. ***Source:******ND Bill HB 1157 (Jan. 2017)***
Other	
Telehealth is a medium to deliver care. OT practitioners must adhere to the same standards as expected for on-site delivery service. Each practitioner must assess and determine if the service delivery method of telehealth meets the standard for each patient encounter using their clinical reasoning and ethical judgment. All legal, regulatory and ethical rules apply consistent with an on-site service. Confidentiality and HIPAA compliance with network connected security in place for video and non-video connections is an important factor. ***Source:******ND Board of Occupational Therapy Practice Practice-Related Information (Aug. 2018).***	No reference found.

**Table:**

Ohio	
Occupational Therapy	Physical Therapy
Telehealth/Telemedicine/Telecommunications Definition	
Telerehabilitation is the clinical application of consultative, preventative, diagnostic, and therapeutic services via two-way interactive telecommunication technology. ***Source:******Ohio Occupational Therapy, Physical Therapy, and Athletic Trainers Board Statement on Telehealth (2010)***	“Telehealth” means the use of electronic communications to provide and deliver a host of health-related information and healthcare services, including, but not limited to physical therapy related information and services, over large and small distances. Telehealth encompasses a variety of healthcare and health promotion activities, including, but not limited to, education, advice, reminders, interventions, and monitoring of interventions. ***Source:******Ohio Admin. Code 4755-27-01***
Modality	
Telehealth includes live video and interactive telecommunication technology. ***Source:******Ohio Occupational Therapy, Physical Therapy, and Athletic Trainers Board Statement on Telehealth (2010)***	Telehealth includes electronic communications and remote monitoring. ***Source:******Ohio Admin. Code 4755-27-01***
Location- Type of site/Geography	
No reference found.	No reference found.
Type of Service	
Occupational therapists may provide individual client services, including evaluation and intervention, via telerehabilitation. ***Source:******Ohio Occupational Therapy, Physical Therapy, and Athletic Trainers Board Statement on Telehealth (2010)***	No reference found.
Supervision	
No reference found.	A supervising physical therapist must be available to a physical therapist assistant by telecommunication at all times, with no requirement for on location supervision. ***Source:******Ohio Admin. Code 4755-27-04.***
Informed Consent	
No reference found.	No reference found.
Patient-Provider-Relationship/In-Person Exam Required	
No reference found.	No reference found.
Licensing	
Occupational therapy personnel must be in possession of a valid Ohio license prior to providing occupational therapy services to clients in Ohio via telerehabilitation. ***Source:******Ohio Occupational Therapy, Physical Therapy, and Athletic Trainers Board Statement on Telehealth (2010)***	If a physical therapy patient is located in Ohio, the physical therapist or physical therapist assistant providing physical therapy services via telehealth must hold a valid license under Ohio statute. ***Source:******Ohio Admin. Code 4755-27-01***
Other	
No reference found.	No reference found.

**Table:**

Oklahoma	
Occupational Therapy	Physical Therapy
Telehealth/Telemedicine/Telecommunications Definition	
No reference found.	No reference found.
Modality	
No reference found.	No reference found.
Location- Type of site/Geography	
No reference found.	No reference found.
Type of Service	
No reference found.	No reference found.
Supervision	
A licensed occupational therapist must be available in person or via telecommunication before implementation of treatment revisions, and to review diagnosis, authorization, client dismissal, and evaluation of treatment with an occupational therapist assistant they are supervising. ***Source:******OK Admin. Code § 435:30-1-16***	General supervision, but not other forms of supervision, of a physical therapist assistant requires the supervising physical therapist to be available by direct telecommunication when not on location. ***Source:******OK Admin. Code § 435:20-1-1(1)***
Informed Consent	
No reference found.	No reference found.
Patient-Provider-Relationship/In-Person Exam Required	
No reference found.	No reference found.
Licensing	
No reference found.	Oklahoma is part of the Physical Therapy Licensure Compact. Physical therapists licensed through the compact may provide services in other compact states and in Oklahoma. ***Source:******Okla. Stat. tit. 59, § 887.1-18***
Other	
No reference found.	No reference found.

**Table:**

Oregon	
Occupational Therapy	Physical Therapy
Telehealth/Telemedicine/Telecommunications Definition	
“Telehealth” is defined as the use of interactive audio and video, in real time telecommunication technology or store-and-forward technology, to deliver health care services when the occupational therapist and patient/client are not at the same physical location. Its uses include diagnosis, consultation, treatment, prevention, transfer of health or medical data, and continuing education. ***Source:******Or. Admin. R. 339-010-0006***	“Telehealth Service” means a physical therapy intervention, including assessment or consultation that can be safely and effectively provided using synchronous two-way interactive video conferencing, or asynchronous video communication, in accordance with generally accepted healthcare practices and standards. For purposes of these rules, “Telehealth service” also means, or may be referred to, as “telepractice, teletherapy, or telerehab” ***Source:******Or. Admin. R. 848-040-0100***
Modality	
Telehealth includes live video and audio or store-and-forward. ***Source:******Or. Admin. R. 339-010-0006***	Telehealth includes live video conferencing or store-and-forward video communication. ***Source:******Or. Admin. R. 848-040-0100***
Location- Type of site/Geography	
No reference found.	No reference found.
Type of Service	
Evaluation or intervention services are allowed as long as the provider considers whether or not the service can be delivered safely via telehealth or if it should take place in person. ***Source:******OR Occupational Therapy Licensing Board, Telehealth FAQs (Aug. 2018)***	Telehealth services must conform to the scope and standard of practice and documentation required in Statute and must be at least equivalent to the quality of services delivered in-person. ***Source:******Or. Admin. R. 848-040-0180***
Supervision	
An occupational therapist may provide routine and general supervision via telehealth, but cannot use telehealth to provide close supervision. ***Source:******Or. Admin. R. 339-010-0006***	A physical therapist must be readily accessible in-person or via telecommunications at all times when a physical therapist assistant is providing physical therapy treatment. ***Source:******Or. Admin. R. 848-015-0020***
Informed Consent	
Occupational therapists are required to obtain informed consent from the patient prior to initiation of occupational therapy services via telehealth and maintain the documentation in the patient’s health record. ***Source:******Or. Admin. R. 339-010-0006***	A patient’s consent must be obtained prior to the initiation of telehealth services. The consent may be verbal, written or recorded and must be documented in the patient’s permanent record. ***Source:******Or. Admin. R. 848-040-0180***
Patient-Provider-Relationship/In-Person Exam Required	
If an in-person intervention is determined to be necessary, every attempt must be made to ensure that an on-site occupational therapist or occupational therapy assistant shall provide the appropriate interventions. In determining whether an in-person evaluation or intervention is necessary, an occupational therapist shall consider, at minimum: The complexity of the patient’s/client’s condition; His or her own knowledge skills and abilities; The patient’s/client’s context and environment; The nature and complexity of the intervention; The pragmatic requirements of the practice setting; and The capacity and quality of the technological interface. ***Source:******Or. Admin. R. 339-010-0006***	No reference found.
Licensing	
To provide services to a client in Oregon, the Occupational Therapist must be licensed by the Oregon OT Licensing Board. Oregon licensed occupational therapists using telehealth to provide services to patients in another state must be licensed in that state. ***Source:******Or. Admin. R. 339-010-0006***	Oregon is part of the Physical Therapy Licensure Compact. Physical therapists licensed through the compact in other compact states may provide services in Oregon. ***Source:******OR SB 1504*** A licensed physical therapist may provide services via telehealth to a patient who is a resident of or who is physically present in the state of Oregon. A physical therapist providing services to a patient not located in Oregon may be required to possess additional licensing. ***Source:******Or. Admin. R. 848-040-0180***
Other	
Telehealth is considered the same as “telepractice” for occupational therapists working in education settings and “teletherapy” and “telerehab” in other settings. An occupational therapist or occupational therapy assistant providing services via telehealth must: Exercise the same standard of care when providing occupational therapy services via telehealth as with any other mode of delivery of occupational therapy services; Provide services consistent with the Standards of Practice; and Comply with provisions of the Occupational Therapy Practice Act and its regulations. ***Source:******Or. Admin. R. 339-010-0006***	When providing telehealth services, the physical therapist must have procedures in place to address remote medical or clinical emergencies at the patient’s location. Technology used to provide telehealth services must meet all standards required by state and federal laws governing the privacy and security of a patient’s protected health information. ***Source:******Or. Admin. R. 848-040-0180***

**Table:**

Pennsylvania	
Occupational Therapy	Physical Therapy
Telehealth/Telemedicine/Telecommunications Definition	
No reference found.	No reference found.
Modality	
No reference found.	Telecommunications may be used for some supervision and to provide consultation. ***Source:******63 Pa. Cons. Stat. § 1302***
Location- Type of site/Geography	
No reference found.	No reference found.
Type of Service	
No reference found.	“Consultation by means of telecommunications” means that a physical therapist renders a professional opinion or advice regarding the practice of physical therapy to another physical therapist or licensed health care provider via telecommunications or computer technology from a distant location. ***Source:******63 Pa. Cons. Stat. § 1302***
Supervision	
No reference found.	A physical therapist is required to be immediately available via telecommunications if not providing direct on-premise supervision to a physical therapist assistant. ***Source:******63 Pa. Cons. Stat. § 1309.1******&******49 Pa. Code § 40.173***
Informed Consent	
No reference found.	No reference found.
Patient-Provider-Relationship/In-Person Exam Required	
No reference found.	No reference found.
Licensing	
No reference found.	A physical therapist holding an unrestricted license in another jurisdiction of the United States may provide consultation via telecommunications without a fee for the consultation. ***Source:******63 Pa. Cons. Stat.§ 1304***
Other	
No reference found.	No reference found.

**Table:**

Rhode Island	
Occupational Therapy	Physical Therapy
Telehealth/Telemedicine/Telecommunications Definition	
Telemedicine is the delivery of healthcare where there is no in-person exchange. ***Source:******RI Department of Health Telemedicine Guidance (Aug. 2018)***	Telemedicine is the delivery of healthcare where there is no in-person exchange. ***Source:******RI Department of Health Telemedicine Guidance (Aug. 2018)***
Modality	
Occupational therapists may use information and communication technologies allowing for the same standards as face-to-face practice. ***Source:******RI Department of Health Telemedicine Guidance (Aug. 2018)***	Physical therapists may use information and communication technologies allowing for the same standards as face-to-face practice. ***Source:******RI Department of Health Telemedicine Guidance (Aug. 2018)***
Location- Type of site/Geography	
No reference found.	No reference found.
Type of Service	
Telemedicine is permitted in the delivery of diagnosis, consultation, treatment, education, care management, and self-management of patients at a distance from health care providers. ***Source:******RI Department of Health Telemedicine Guidance (Aug. 2018)***	Telemedicine is permitted in the delivery of diagnosis, consultation, treatment, education, care management, and self-management of patients at a distance from health care providers. ***Source:******RI Department of Health Telemedicine Guidance (Aug. 2018)***
Supervision	
No reference found.	A physical therapist providing supervision to a physical therapy assistant must be available at all times via telecommunications while the physical therapy assistant is providing services to patients. ***Source:******216 40 R.I. Code R. § 05-13***
Informed Consent	
Patient informed consent is required for the use of patient-physician e-mail and other text based communication. The agreement should be discussed with the patient and should include the following terms: Types of transmissions that will be permitted (prescription refills, appointment scheduling, patient education, etc.); Circumstances when alternate forms of communication or office visits should be utilized; Security measures, such as encryption of data, password protected screen savers and data files, or utilization of other reliable authentication techniques, as well as potential risks to privacy; Hold harmless clause for information lost due to technical failures; Requirement for express patient consent to forward patient-identifiable information to a third party; and A statement noting that the patient’s failure to comply with the agreement may result in the physician terminating the e-mail relationship. ***Source:******RI Department of Health Telemedicine Guidance (Aug. 2018)***	Patient informed consent is required for the use of patient-physician e-mail and other text based communication. The agreement should be discussed with the patient and should include the following terms: Types of transmissions that will be permitted (prescription refills, appointment scheduling, patient education, etc.); Circumstances when alternate forms of communication or office visits should be utilized; Security measures, such as encryption of data, password protected screen savers and data files, or utilization of other reliable authentication techniques, as well as potential risks to privacy; Hold harmless clause for information lost due to technical failures; Requirement for express patient consent to forward patient-identifiable information to a third party; and A statement noting that the patient’s failure to comply with the agreement may result in the physician terminating the e-mail relationship. ***Source:******RI Department of Health Telemedicine Guidance (Aug. 2018)***
Patient-Provider-Relationship/In-Person Exam Required	
A documented patient evaluation, including history and physical evaluation must be obtained prior to providing treatment electronically or otherwise. Treatment based only on an online questionnaire without appropriate evaluation does not constitute as an acceptable standard of care and is considered unprofessional conduct. ***Source:******RI Department of Health Telemedicine Guidance (Aug. 2018)***	A documented patient evaluation, including history and physical evaluation must be obtained prior to providing treatment electronically or otherwise. Treatment based only on an online questionnaire without appropriate evaluation does not constitute as an acceptable standard of care and is considered unprofessional conduct. ***Source:******RI Department of Health Telemedicine Guidance (Aug. 2018)***
Licensing	
No reference found.	No reference found.
Other	
A patient’s medical records should include patient-related electronic communications, including patient-physician e-mail, prescriptions, laboratory and test results, evaluations and consultations, records of past care and instructions pertinent to the diagnosis and treatment of the patient. Occupational therapists should meet or exceed federal and state legal requirements of medical/health information privacy. Occupational therapy practice sites should clearly disclose: Owner of the site; Specific services provided; Office addresses and contact information; Licensure and qualifications of physician(s) and associated healthcare providers; Fees for online consultation and services and how payment is to be made; Financial interests in any information, products or services; Appropriate uses and limitations of the site, including providing health advice and emergency health situations; Uses and response times for e-mails, electronic messages and other communications transmitted via the site; To whom patient health information may be disclosed and for what purpose; Rights of patients with respect to patient health information; Information collected and any passive tracking mechanisms utilized. ***Source:******RI Department of Health Telemedicine Guidance (Aug. 2018)***	A patient’s medical records should include patient-related electronic communications, including patient-physician e-mail, prescriptions, laboratory and test results, evaluations and consultations, records of past care and instructions pertinent to the diagnosis and treatment of the patient. Physical therapists should meet or exceed federal and state legal requirements of medical/health information privacy. Physical therapy practice sites should clearly disclose: Owner of the site; Specific services provided; Office addresses and contact information; Licensure and qualifications of physician(s) and associated healthcare providers; Fees for online consultation and services and how payment is to be made; Financial interests in any information, products or services; Appropriate uses and limitations of the site, including providing health advice and emergency health situations; Uses and response times for e-mails, electronic messages and other communications transmitted via the site; To whom patient health information may be disclosed and for what purpose; Rights of patients with respect to patient health information; Information collected and any passive tracking mechanisms utilized. ***Source:******RI Department of Health Telemedicine Guidance (Aug. 2018)***

**Table:**

South Carolina	
Occupational Therapy	Physical Therapy
Telehealth/Telemedicine/Telecommunications Definition	
Telehealth, also known as telemedicine, is the provision of health care, health information, and health education across a distance, using telecommunications technology and specially adapted equipment. Telehealth physicians, nurses and health care specialist assess, diagnose, and treat patients without requiring individuals to be physically located in the same place, regardless of whether that distance is across the street, across a city, across state lines or across continents. ***Source:******SC OT Board eNews (Apr. 2010)***	No reference found.
Modality	
Face-to-face and store-and-forward are recognized and permitted by South Carolina for services and supervision. ***Source:******SC OT Board eNews (Apr. 2010)***	No reference found.
Location- Type of site/Geography	
Telehealth is practiced in many settings such as rural hospitals, school districts, home health settings, nursing homes, cruise ships, battle fields and even NASA space missions. Occupational therapy must be licensed in the state in which the patient is receiving their services. Teleconsultations between providers may have different laws depending on which state the providers are located in. ***Source:******SC OT Board eNews (Apr. 2010)***	No reference found.
Type of Service	
No reference found.	No reference found.
Supervision	
Supervision requirements from the Occupational Therapy Practice Act should be followed if providing supervision via telehealth. ***Source:******SC OT Board eNews (Apr. 2010)***	No reference found.
Informed Consent	
Occupational therapists should receive informed consent and allow patients the opportunity to refuse the telehealth service. ***Source:******SC OT Board eNews (Apr. 2010)***	No reference found.
Patient-Provider-Relationship/In-Person Exam Required	
No reference found.	No reference found.
Licensing	
Occupational therapists must be licensed in the state where the patient is located when providing telehealth services. ***Source:******SC OT Board eNews (Apr. 2010)***	South Carolina is part of the Physical Therapy Licensure Compact. Physical therapists licensed through the compact in other compact states may provide services in South Carolina. ***Source:******S.C. Code Ann. § 40-45-520***
Other	
Occupational therapists providing services via telehealth should apply best practices and competencies related to service delivery, operating hardware and software systems, and access to technical support. ***Source:******SC OT Board eNews (Apr. 2010)***	No reference found.

**Table:**

South Dakota	
Occupational Therapy	Physical Therapy
Telehealth/Telemedicine/Telecommunications Definition	
No reference found.	No reference found.
Modality	
No reference found.	Telecommunications, which would include live video and audio and telephone, may be used for some supervision. ***Source:******S.D. Codified Laws § 36-10-35.9***
Location- Type of site/Geography	
No reference found.	No reference found.
Type of Service	
No reference found.	No reference found.
Supervision	
No reference found.	A supervising physical therapist must be accessible to a physical therapist assistant either in person or via telecommunications at all times the physical therapist assistant is treating a patient. ***Source:******S.D. Codified Laws § 36-10-35.9***
Informed Consent	
No reference found.	No reference found.
Patient-Provider-Relationship/In-Person Exam Required	
No reference found.	No reference found.
Licensing	
No reference found.	No reference found.
Other	
No reference found.	No reference found.

**Table:**

Tennessee	
Occupational Therapy	Physical Therapy
Telehealth/Telemedicine/Telecommunications Definition	
“Telehealth” or ‘telemedicine’ means the use of real-time audio, video, or other electronic media and telecommunications technologies that enable interaction between the healthcare provider and the patient, or also store-and-forward telemedicine services for the purpose of diagnosis, consultation, or treatment of a patient in another location where there may be no in-person exchange. ***Source:******Tenn. Code Ann. § 63-1-155***	“Telehealth” or ‘telemedicine’ means the use of real-time audio, video, or other electronic media and telecommunications technologies that enable interaction between the healthcare provider and the patient, or also store-and-forward telemedicine services for the purpose of diagnosis, consultation, or treatment of a patient in another location where there may be no in-person exchange. ***Source:******Tenn. Code Ann. § 63-1-155***
Modality	
Telehealth includes live audio, video, or other electronic media and store-and-forward services. ***Source:******Tenn. Code Ann. § 63-1-155***	Telehealth includes live audio, video, or other electronic media and store-and-forward services. ***Source:******Tenn. Code Ann. § 63-1-155***
Location- Type of site/Geography	
No reference found.	A physical therapist may not provide remote supervision to a physical therapist assistant while more than 60 miles or one hour away from the physical therapist assistant. ***Source:******Tenn. Comp. R & Regs. 1150-01-.02***
Type of Service	
Telehealth may be used to provide diagnosis, consultation, or treatment of a patient. ***Source:******Tenn. Code Ann. § 63-1-155*** Phone and electronic communication may be used for some supervision. ***Source:******Tenn. Comp. R & Regs. 1150-02***	Telehealth may be used to provide diagnosis, consultation, or treatment of a patient. ***Source:******Tenn. Code Ann. § 63-1-155*** Consultation means a meeting that is conducted either face-to-face or by some other medium such as, but not limited to, telephone, facsimile, mail, or electronic means, wherein two or more health professionals discuss the diagnosis, prognosis, and treatment of a particular case. ***Source:******Tenn. Comp. R & Regs. 1150-01-.01***
Supervision	
Methods of supervision include but are not limited to: In person Phone contact Electronic contact ***Source:******Tenn. Comp. R & Regs. 1150-02***	When a physical therapist assistant is practicing in an offsite setting, the supervising physical therapist must be immediately available via telecommunications. ***Source:******Tenn. Code Ann. § 63-13-103******&******Tenn. Comp. R & Regs. 1150-01***
Informed Consent	
An occupational therapist may obtain either expressed or implied consent. ***Source:******Tenn. Code Ann. § 63-1-155***	A physical therapist may obtain either expressed or implied consent. ***Source:******Tenn. Code Ann. § 63-1-155***
Patient-Provider-Relationship/In-Person Exam Required	
A provider-patient relationship is established upon creating mutual consent and mutual communication. ***Source:******Tenn. Code Ann. § 63-1-155***	A provider-patient relationship is established upon creating mutual consent and mutual communication. ***Source:******Tenn. Code Ann. § 63-1-155***
Licensing	
Occupational therapists must be licensed in Tennessee if providing services to a patient located within the state. ***Source:******Tenn. Code Ann. § 63-1-155***	Tennessee is part of the Physical Therapy Licensure Compact. Physical therapists licensed through the compact in other compact states may provide services in Tennessee. ***Source:******Tenn. Code Ann. § 63-13-402***
Other	
Occupational therapists are held to the same standard of care as in-person occupational therapy. ***Source:******Tenn. Code Ann. § 63-1-155***	Physical therapists are held to the same standard of care as in-person physical therapy. ***Source:******Tenn. Code Ann. § 63-1-155***

**Table:**

Texas	
Occupational Therapy	Physical Therapy
Telehealth/Telemedicine/Telecommunications Definition	
Telehealth is a mode of service delivery for the provision of occupational therapy services delivered by an occupational therapy practitioner to a client at a different physical location using telecommunications or information technology. Telehealth refers only to the practice of occupational therapy by occupational therapy practitioners who are licensed by this Board with clients who are located in Texas at the time of the provision of occupational therapy services. Also may be known as other terms including but not limited to telepractice, telecare, telerehabilitation, and e-health services. ***Source:******40 Tex. Admin. Code § 362.1***	No reference found.
Modality	
An occupational therapist must have contact with a client during an intervention via live audio-visual telehealth or in-person. Other telecommunications may be used to aid in the intervention, but may not be the primary means of communication. ***Source:******40 Tex. Admin. Code § 372.1***	No reference found.
Location- Type of site/Geography	
No reference found.	No reference found.
Type of Service	
Telehealth may be used for occupational therapy services. ***Source:******40 Tex. Admin. Code § 362.1***	No reference found.
Supervision	
Up to half of the required hours of interactive supervision of occupational therapy assistants may be conducted via synchronous electronic communication technologies. Frequent communication supervision may include electronic or communications technology methods. ***Source:******40 Tex. Admin. Code § 373.3*** Supervision of a non-licensed personnel other than an occupational therapy aide provided either on-site or via telehealth, requires the occupational therapy practitioner to maintain line of sight. ***Source:******40 Tex. Admin. Code § 373.1***	No reference found.
Informed Consent	
No reference found.	No reference found.
Patient-Provider-Relationship/In-Person Exam Required	
No reference found.	No reference found.
Licensing	
Occupational therapists must be licensed in Texas if providing telehealth services to patients located within the state. ***Source:******40 Tex. Admin. Code § 362.1***	Texas is part of the Physical Therapy Licensure Compact. Physical therapists licensed through the compact in other compact states may provide services in Texas. ***Source:******Tex. Occ. Code § 453.501***
Other	
An occupational therapist is responsible for determining whether an evaluation may be conducted via telehealth or must be conducted in person. ***Source:******40 Tex. Admin. Code § 372.1***	No reference found.

**Table:**

Utah	
Occupational Therapy	Physical Therapy
Telehealth/Telemedicine/Telecommunications Definition	
No reference found.	No reference found.
Modality	
No reference found.	Use of telephone or electronic communication is allowed for some supervision. ***Source:******Utah Code § 58 24b-102***
Location- Type of site/Geography	
No reference found.	No reference found.
Type of Service	
No reference found.	“Consultation by telecommunication” means the provision of expert or professional advice by a physical therapist who is licensed outside of Utah to a licensed physical therapist or a health care provider by telecommunication or electronic communication. ***Source:******Utah Code § 58 24b-102***
Supervision	
Occupational therapists and occupational therapy assistants are required to participate in biweekly face-to-face meetings which may be held via videoconference. ***Source:******Utah Code § 58 42a-306***	“General supervision” means supervision and oversight of a person by a licensed physical therapist when the licensed physical therapist is immediately available in person, by telephone, or by electronic communication to assist the person. ***Source:******Utah Code § 58 24b-102***
Informed Consent	
No reference found.	No reference found.
Patient-Provider-Relationship/In-Person Exam Required	
No reference found.	No reference found.
Licensing	
No reference found.	Utah is part of the Physical Therapy Licensure Compact. Physical therapists licensed through the compact in other compact states may provide services in Utah. ***Source:******Utah Code § 58 24c-101*** A person without a Utah physical therapy license may provide consultation via telecommunication to a physical therapist in Utah. ***Source:******Utah Code § 58 24b-304***
Other	
No reference found.	No reference found.

**Table:**

Vermont	
Occupational Therapy	Physical Therapy
Telehealth/Telemedicine/Telecommunications Definition	
No reference found.	No reference found.
Modality	
Telephone or electronic communication may be used for some supervision. ***Source:******4 Vt. Code R. 030 190***	Audio, video, or data communications may be used to provide a distance consultation. ***Source:******Vt. Stat. Ann. tit. 26, § 2081a*** Telecommunications, which would include live video and audio and telephone, may be used in some supervision. ***Source:******4 Vt. Code R. 030 240 2.1***
Location- Type of site/Geography	
No reference found.	No reference found.
Type of Service	
No reference found.	“Distance consultation” means the rendering of professional or expert opinion or advice to a licensed physical therapist, including the review or transfer of patient records or related information by means of audio, video, or data communications. ***Source:******Vt. Stat. Ann. tit. 26, § 2081a***
Supervision	
Routine supervision requires interim supervision which may be provided to an occupational therapy assistant via telephonic, electronic, or written communication. ***Source:******4 Vt. Code R. 030 190***	General supervision requires that the licensed physical therapist be available at least by telecommunications. ***Source:******4 Vt. Code R. 030 240 2.1***
Informed Consent	
No reference found.	No reference found.
Patient-Provider-Relationship/In-Person Exam Required	
No reference found.	No reference found.
Location	
No reference found.	No reference found.
Other	
No reference found.	No reference found.

**Table:**

Virginia	
Occupational Therapy	Physical Therapy
Telehealth/Telemedicine/Telecommunications Definition	
No reference found.	Telehealth is the use of electronic technology or media including interactive audio or video to engage in the practice of physical therapy. Telehealth does not include an audio-only telephone, electronic mail message, facsimile transmission, or online questionnaire. ***Source:******Board of Physical Therapy Guidance on Telehealth (2015)***
Modality	
No reference found.	Telehealth includes the use of live video. It does not include audio-only telephone, electronic mail message, facsimile transmission, or online questionnaire. ***Source:******Board of Physical Therapy Guidance on Telehealth (2015)***
Location- Type of site/Geography	
No reference found.	No reference found.
Type of Service	
No reference found.	Telehealth may be used to provide physical therapy services. ***Source:******Board of Physical Therapy Guidance on Telehealth (2015)***
Supervision	
No reference found.	No reference found.
Informed Consent	
No reference found.	Documented consent is required prior to the delivery of telehealth services. Clients should be made aware of limitations surrounding telehealth services. The documentation of consent should include: Identification of the client, practitioner, and practitioner’s credentials; Types of activities permitted using telehealth services; Details on security measures taken and potential risks to privacy notwithstanding such measures; A hold harmless clause for information lost due to technical failures; and Requirements for express client consent to forwards client-identifiable information to a third party ***Source:******Board of Physical Therapy Guidance on Telehealth (2015)***
Patient-Provider-Relationship/In-Person Exam Required	
No reference found.	A physical therapist-client relationship can be established in the absence of actual physical contact between the physical therapist and client. Just as in a traditional (in-person) encounter, once the relationship is established, the therapist has an obligation to adhere to the reasonable standards of care for the client (duty of care). ***Source:******Board of Physical Therapy Guidance on Telehealth (2015)***
License	
No reference found.	Physical therapist must be licensed in the state where the patient is located and where the therapist is located when providing telehealth services. ***Source:******Board of Physical Therapy Guidance on Telehealth (2015)***
Other	
No reference found.	Physical therapists are responsible for determining if telehealth use is appropriate for the situation. The identities of both the client and the practitioner should be verified with photo IDs. The documentation of telehealth sessions should: Be at least up to standards on in-person care, and additionally include specifications of telehealth technology used. Include written policies and procedures for documentation and confidentiality of identifiable client health information and records of encounters using telehealth. Include procedures to address technical, medical, or clinical emergencies at the remote destination need to be in place. ***Source:******Board of Physical Therapy Guidance on Telehealth (2015)***

**Table:**

Washington	
Occupational Therapy	Physical Therapy
Telehealth/Telemedicine/Telecommunications Definition	
Telehealth means providing occupational therapy via electronic communication where the occupational therapist or occupational therapy assistant and the patient are not at the same physical location. ***Source:******Wash. Admin. Code § 246-847-176***	Telehealth means providing physical therapy via electronic communication where the physical therapist or physical therapist assistant and the patient are not at the same physical location. Electronic communication means the use of interactive, secure multimedia equipment that includes, at a minimum, audio and video equipment permitting two-way, real time interactive communication between the physical therapist or the physical therapist assistant and the patient. ***Source:******Wash. Admin. Code § 246-915-187***
Modality	
No reference found.	Telehealth includes the use of live audio and video. ***Source:******Wash. Admin. Code § 246-915-187***
Location- Type of site/Geography	
No reference found.	No reference found.
Type of Service	
Telehealth may be used to delivery occupational therapy services. ***Source:******Wash. Admin. Code § 246-847-176***	Telehealth may be used to provide physical therapy services. ***Source:******Wash. Admin. Code § 246-915-187***
Supervision	
No reference found.	No reference found.
Informed Consent	
No reference found.	No reference found.
Patient-Provider-Relationship/In-Person Exam Required	
No reference found.	No reference found.
Licensing	
Occupational therapists must be licensed in Washington if providing telehealth services to a patient located within the state. ***Source:******Wash. Admin. Code § 246-847-176***	Washington is part of the Physical Therapy Licensure Compact. Physical therapists licensed through the compact in other compact states may provide services in Washington. ***Source:******Wash. Rev. Code § 18 74-500***
Other	
Licensed occupational therapists and occupational therapy assistants may provide therapy via telehealth if all requirements for supervision and standards of care are met. Use of telehealth must be identified in the clinical record. ***Source:******Wash. Admin. Code § 246-847-176***	Licensed physical therapists and physical therapy assistants providing physical therapy services via telehealth must follow the standards of care defined in the Washington Administrative Code. ***Source:******Wash. Rev. Code § 18 74*** A provider must document when a physical therapy service is provided via telehealth. ***Source:******Wash. Admin. Code § 246-915-187***

**Table:**

West Virginia	
Occupational Therapy	Physical Therapy
Telehealth/Telemedicine/Telecommunications Definition	
No reference found.	Telecommunication means audio, video, or data communication. ***Source:******W. Va. Code § 30-20-3***
Modality	
Electronic communication, which would include live video and audio and telephone, may be used for some supervision. ***Source:******W. VA. Code R. § 13-1-2***	A physical therapist is permitted to use audio, video, or data communication. ***Source:******W. Va. Code § 30-20-3*** Telecommunications, which would include live video and audio and telephone, may be used for some supervision. ***Source:******W. Va. Code § 30-20-3******&******W. VA. Code R. § 1316-1-2***
Location- Type of site/Geography	
No reference found.	No reference found.
Type of Service	
No reference found.	“Consultation” means a physical therapist renders an opinion or advice to another physical therapist or health care provider through telecommunications. ***Source:******W. VA. Code R. § 16-1-2***
Supervision	
An occupational therapist may provide general supervision to an occupational therapy assistant via electronic communication. Electronic communication is not applicable to direct supervision, direct close supervision, or direct continuous supervision. ***Source:******W. VA. Code R. § 13-1-2***	A physical therapist must be available at least via telecommunications when providing general supervision. ***Source:******W. Va. Code § 30-20-3******&******W. VA. Code R. § 16-******1-2*** Physical therapist must be available to make a joint onsite visit with the physical therapist assistant within 24 hours as prudent practice indicates. ***Source:******W. VA. Code R. § 16-1-8***
Informed Consent	
No reference found.	No reference found.
Patient-Provider-Relationship/In-Person Exam Required	
No reference found.	No reference found.
Licensing	
No reference found.	West Virginia is part of the Physical Therapy Licensure Compact. Physical therapists licensed through the compact in other compact states may provide services in West Virginia. ***Source:******W. Va. Code § 30-41-1***
Other	
No reference found.	No reference found.

**Table:**

Wisconsin	
Occupational Therapy	Physical Therapy
Telehealth/Telemedicine/Telecommunications Definition	
No reference found.	No reference found.
Modality	
Telephone, electronic communication, or group conferencing may be used in some supervision. ***Source:******Wis. Admin. Code OT. § 4.04***	Telecommunications, which would include live video and audio and telephone, may be used for some supervision. ***Source:******Wis. Admin. Code PT. 1.02******&******Wis. Admin. Code PT. 5.01***
Location- Type of site/Geography	
No reference found.	No reference found.
Type of Service	
No reference found.	No reference found.
Supervision	
While providing supervision to an occupational therapy assistant, the supervising occupational therapist is required to provide direct contact and face-to-face contact which may be accomplished via telephone, electronic communication, or group conference. ***Source:******Wis. Admin. Code OT. § 4.04***	A physical therapist may provide off-site, supplemental general supervision via telecommunications. “General supervision” means direct, on-premises contact between a supervisor, and a physical therapist, physical therapist assistant, student or temporary licensee being supervised, as necessary. Between direct contacts, a supervisor is required to maintain indirect, off-premises telecommunication contact such that the person being supervised can, within 24 hours, establish direct telecommunication with a supervisor. ***Source:******Wis. Admin. Code PT. 1.02*** A physical therapist must have direct face-to-face contact with the physical therapist assistant at least every 14 calendar days, unless the board approves another type of contact. They also must remain accessible to telecommunication in the interim. ***Source:******Wis. Admin. Code PT. 5.01***
Informed Consent	
No reference found.	No reference found.
Patient-Provider-Relationship/In-Person Exam Required	
No reference found.	No reference found.
Licensing	
No reference found.	No reference found.
Other	
No reference found.	No reference found.

**Table:**

Wyoming	
Occupational Therapy	Physical Therapy
Telehealth/Telemedicine/Telecommunications Definition	
Occupational therapy telehealth means the provision of occupational therapy services across a distance, using telecommunications technology for the evaluation, intervention or consultation without requiring the occupational therapist and recipient to be physically located in the same place. ***Source:******Wyo. Stat. Ann. § 33.40***	No reference found.
Modality	
Telehealth includes the use of telecommunications, which would include live video and audio and telephone, for the delivery of services and for some supervision. ***Source:******Wyo. Stat. Ann. § 33.40******&******083-0001 Wyo. Code R. § 1***	Telecommunications and computer technology include the use of audio, video, or data communications. ***Source:******062-0001 Wyo. Code R. § 1***
Location- Type of site/Geography	
No reference found.	No reference found.
Type of Service	
The tasks of occupational therapy, including evaluation, intervention or consultation, may be delivered via telecommunication services and other communication technologies. An occupational therapist may use telehealth to provide and receive consultation from another occupational therapist. ***Source:******Wyo. Stat. Ann. § 33.40***	A physical therapist may use telehealth for services that are legally or professionally authorized. ***Source:******062-0001 Wyo. Code R. § 1*** A physical therapist may provide professional consultation to another physical therapist via telecommunications or other computer technology from a distant location. ***Source:******062-0001 Wyo. Code R. § 1*** “Consultation using telecommunication” means the provision of professional or expert opinion or advice to a physical therapist or other health care provider using telecommunication or computer technology from a distant location. It includes the review or transfer of patient records or related information using audio, video or data communications. ***Source:******Wyo. Stat. Ann. § 33.25-101*** Consultation by means of telecommunications means that a physical therapist renders professional or expert opinion or advice to another physical therapist or healthcare provider via telecommunications or computer technology from a distant location. It includes the transfer of data or exchange of educational or related information by means of audio, video, or data communications. The physical therapist may use telehealth technology as a vehicle for providing only services that are legally or professionally authorized. ***Source:******062-0001 Wyo. Code R. § 1***
Supervision	
An occupational therapist may provide general or routine supervision via electronic communication. ***Source:******083-0001 Wyo. Code R. § 1***	A supervising physical therapist must be available at all times for consultation with a physical therapist assistant either in person or via telecommunications. ***Source:******062-0001 Wyo. Code R. § 7***
Informed Consent	
No reference found.	The patient’s written or verbal consent must be obtained and documented prior to a consultation via telecommunications. ***Source:******062-0001 Wyo. Code R. § 1***
Patient-Provider-Relationship/In-Person Exam Required	
No reference found.	No reference found.
Licensing	
No reference found.	A physical therapist providing consultation via telecommunication is exempt from the Wyoming licensure requirements. ***Source:******Wyo. Stat. Ann. § 33.25-102***
Other	
No reference found.	All records resulting from a telecommunications consultation must be recorded as part of a patient’s record. ***Source:******062-0001 Wyo. Code R. § 1***
